# Ablation of kynurenine 3-monooxygenase rescues plasma inflammatory cytokine levels in the R6/2 mouse model of Huntington’s disease

**DOI:** 10.1038/s41598-021-84858-7

**Published:** 2021-03-09

**Authors:** Marie Katrin Bondulich, Yilan Fan, Yeojin Song, Flaviano Giorgini, Gillian P. Bates

**Affiliations:** 1 Huntington’s Disease Centre, Department of Neurodegenerative Disease and UK Dementia Research Institute At UCL, Queen Square Institute of Neurology, UCL, Queen Square, WC1N 3BG UK; 2 Department of Genetics and Genome Biology, University of Leicester, Leicester, LE1 7RH UK

**Keywords:** Neuroscience, Diseases of the nervous system, Neuroimmunology

## Abstract

Kynurenine 3-monooxygenase (KMO) regulates the levels of neuroactive metabolites in the kynurenine pathway (KP), dysregulation of which is associated with Huntington’s disease (HD) pathogenesis. KMO inhibition leads to increased levels of neuroprotective relative to neurotoxic metabolites, and has been found to ameliorate disease-relevant phenotypes in several HD models. Here, we crossed KMO knockout mice to R6/2 HD mice to examine the effect of KMO depletion in the brain and periphery. KP genes were dysregulated in peripheral tissues from R6/2 mice and KMO ablation normalised levels of a subset of these. KP metabolites were also assessed, and KMO depletion led to increased levels of neuroprotective kynurenic acid in brain and periphery, and dramatically reduced neurotoxic 3-hydroxykunurenine levels in striatum and cortex. Notably, the increased levels of pro-inflammatory cytokines TNFa, IL1β, IL4 and IL6 found in R6/2 plasma were normalised upon KMO deletion. Despite these improvements in KP dysregulation and peripheral inflammation, KMO ablation had no effect upon several behavioural phenotypes. Therefore, although genetic inhibition of KMO in R6/2 mice modulates several metabolic and inflammatory parameters, these do not translate to improvements in primary disease indicators—observations which will likely be relevant for other interventions targeted at peripheral inflammation in HD.

## Introduction

Huntington’s disease (HD) is a devastating inherited neurodegenerative disease that is characterised by progressive clinical features including motor dysfunction, cognitive decline and psychiatric disturbances^1^. The disease is caused by the expansion of an unstable CAG trinucleotide repeat within exon 1 of the huntingtin gene (*HTT*). This results in an abnormally long polyglutamine (polyQ) tract in the huntingtin protein (HTT)^2^. The *HTT* CAG repeat is polymorphic in the normal population with a range of 6–35 units, for those with 40 or more the disease will inevitably manifest within a normal lifespan, and with individuals carrying 36–39 CAGs having an increased risk of developing HD^3^. Furthermore, repeat sizes of more than 65 can cause the juvenile form of the disease^4^. HD neuropathology is characterised by huntingtin-rich aggregates, widespread atrophy, pronounced cell loss and neuronal dysfunction in the striatum, cortex and other brain regions^5^. However, HD is increasingly recognised as a disease of both the CNS and periphery with specific aspects of the disease likely being mediated outside the CNS^6^.

**Figure 1:**
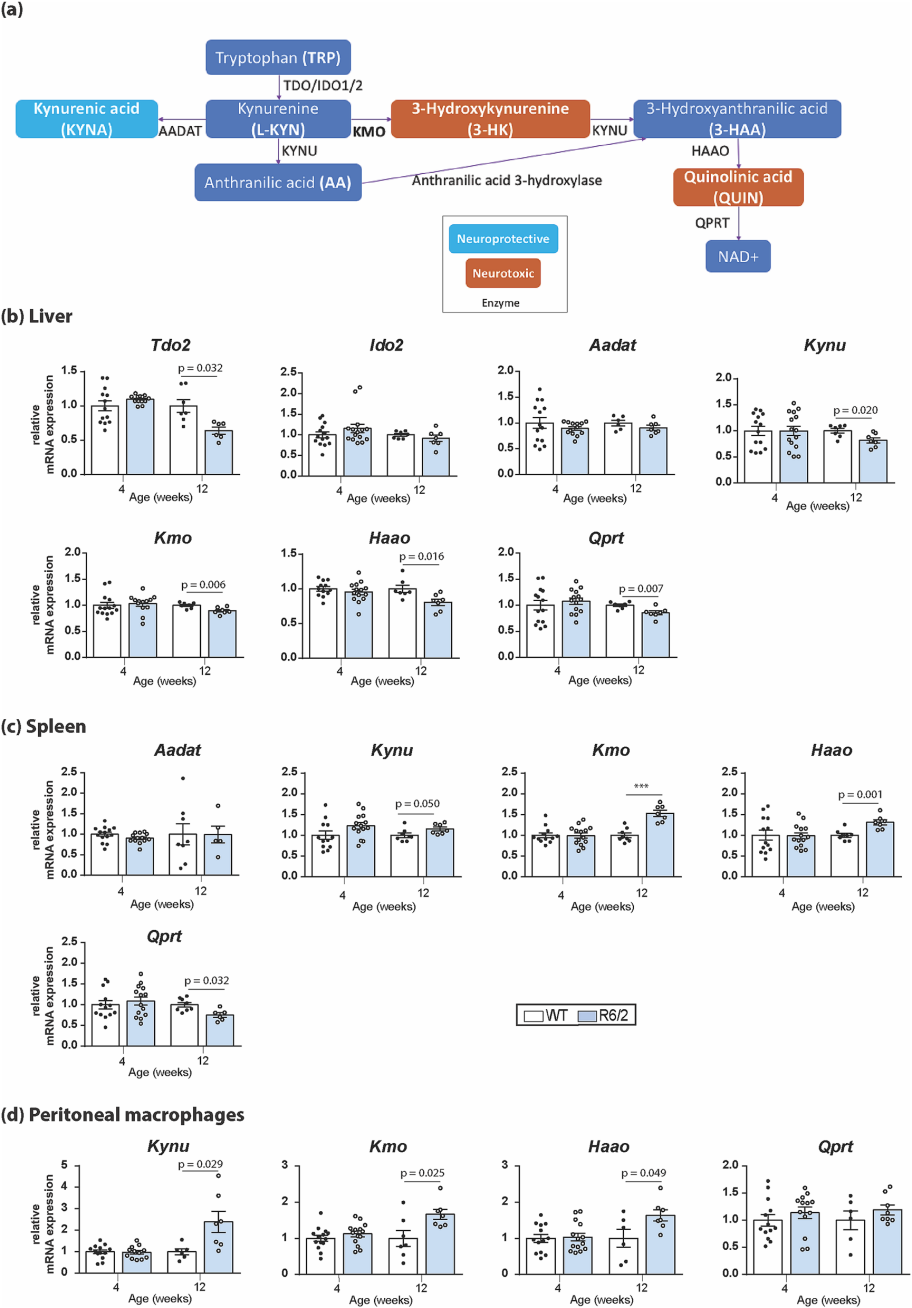
Transcript levels of KP pathway enzymes in late-stage R6/2 liver, spleen and peritoneal macrophages. (**a**) Simplifed schematic representation of the metabolites and enzymes of the KP. In the context of neurodegeneration, the KP features a key branching point for the metabolism of L-KYN. KMO converts L-KYN into the neurotoxic, free-radical generator 3-HK, which leads to the further synthesis of 3-HAA and the neurotoxic metabolite QUIN. L-KYN can also be metabolized by the aminoadipate aminotransferase family of enzymes to produce KYNA, which conveys neuroprotection. (**b**) Comparison of the levels of liver KP transcripts, as measured by qPCR, between 4 and 12 weeks old R6/2 and WT littermates. Transcript levels for *Tdo2*, *Kynu*, *Kmo*, *Haao* and *Qprt* were all reduced in R6/2 compared to WT littermates at 12 weeks of age. (**c**) Comparison of the levels of spleen transcripts, as measured by qPCR, between 4 and 12 weeks old R6/2 and WT littermates. Transcript levels for *Kynu*, *Kmo* and *Haao* were elevated and *Qprt* was reduced in R6/2 compared to WT littermates at 12 weeks of age. (**d**) Comparison of the levels of peritoneal macrophages transcripts, as measured by qPCR, between 4 and 12 weeks old R6/2 and WT littermates. Transcript levels for *Kynu*, *Kmo* and *Haao* were all elevated in R6/2 compared to WT littermates at 12 weeks of age. Statistical analyses via Student’s *t*-test; *p*-values displayed in graph, n = 8 per gender/genotype. The test statistic, degrees of freedom and *p* values for the *t*-test are provided in Supplementary Table S7 online. WT = wild type.

The kynurenine pathway (KP) has been closely linked to the pathogenesis of several neurodegenerative disorders including HD. The KP is responsible for > 95% of tryptophan degradation in mammals, ultimately leading to the production of nicotinamide adenosine dinucleotide (NAD^+^). It contains three well-characterized neuroactive metabolites: kynurenic acid (KYNA), 3-hydroxykynurenine (3-HK), and quinolinic acid (QUIN) (Fig. 1a)^7^. QUIN is an excitotoxic N-methyl-D-aspartate (NMDA) receptor agonist which can potently stimulate lipid peroxidation and generate reactive oxygen species (ROS) when complexed with ferrous iron, while 3-HK is a potent free-radical generator^8^. On the other hand, KYNA, has been shown to be neuroprotective, acting as an antagonist of excitatory amino acid receptors and a free-radical scavenger^9^. Imbalance of neurotoxic and neuroprotective KP metabolites is closely linked to the pathogenesis of HD, with increased flux through the central pathway producing 3-HK and QUIN, combined with a reduced flux through the protective KYNA producing arm^7^. The KP enzyme kynurenine 3-monooxygenase (KMO) lies at this branch point and thereby regulates the balance between neurotoxic and neuroprotective metabolites. Notably, pharmacological and genetic inhibition of KMO has been found to normalise this metabolic imbalance and ameliorate disease phenotypes in several models of HD^10–13^.

Chronic activation of both the peripheral and central nervous system have been reported in HD^14–16^. Previous studies, including ones in our own lab, have shown pro- and anti-inflammatory cytokines such as TNFα, IL2, IL1β, IL6 and IL10 to be elevated in HD blood and brain during the course of the disease^15–17^. Although relatively little is known about the interaction between KMO and the innate immune system in vivo, emerging data indicates that macrophage/cytokine influx can cause significant changes to the levels and ratios of KP metabolites in the CNS, and that enzymes within the KMO branch of KP may be induced by proinflammatory stimuli^18^. Similarly, in a systemic inflammatory rat model induced by lipopolysaccharide (LPS), an increase in the level of KMO transcripts was observed in both cortex and hippocampus one day post LPS administration^19^. In the neuroprotective KP branch, *Aadat* expression is either unaffected or decreased by proinflammatory stimuli, depending on the model used and the subtypes of *Aadat* studied^18^.

Here we demonstrate that genetic ablation of KMO in the R6/2 mouse model of HD modulates the concentrations of KP metabolites in the CNS and the periphery without causing any adverse effects. This restored the transcript levels of KP enzymes that are distal to KMO, and reduced the plasma levels of several proinflammatory cytokines that are elevated in R6/2 mice. Therefore, our results suggest that the inhibition of KMO may target HD phenotypes caused by dysregulation of the immune system. Although several pharmacological inhibitors of KMO have demonstrated selective beneficial effects when used for treating HD mice^11,12,20^, consistent with these studies, we found that genetic ablation of KMO had did not improve behavioural or phenotypic deficits.

## Results

### KP pathway enzymes are transcriptionally dysregulated in the liver, spleen and peritoneal macrophages of late-stage R6/2 mice

Levels of KP metabolites have previously been found to be dysregulated in the brains of HD mice^21,22^. Given that transcriptional dysregulation is widespread in HD, this could be due to altered expression of KP pathway enzymes (Fig. 1a). Therefore, we assessed transcript levels of KP enzymes in early (4 weeks) and late stage (12 weeks) R6/2 mice by qPCR. While the KP enzymes were expressed at levels too low for detection in the cortex and striatum of all animals tested (Supplementary Table S1 online), robust expression was found for all the pathway enzymes in liver (Supplementary Table S2 online). A statistically significant reduction of *Tdo2, Kynu, Kmo, Haao* and *Qprt* expression was detected at 12 weeks of age in R6/2 liver in comparison to wild type (WT) littermates, with *Tdo* expression showing the most pronounced decrease (~ 39% of WT levels) (Fig. 1b). In contrast to the liver, the levels of *Kynu, Kmo and Haao* were increased in R6/2 spleen at 12 weeks of age (Fig. 1c), with *Kmo* showing the greatest change (1.5 fold increase), whereas *Qprt* was decreased, as observed in the liver (Fig. 1b). It was not possible to detect the expression of either *Tdo2* or *Ido2* in the spleen where low levels of *Aadat* were also observed (Supplementary Table S3 online). The level of RNA extracted from peritoneal macrophages was very low, making it difficult to detect the expression of all KP enzymes (Supplementary Table S4 online). Nonetheless, *Haao, Kmo* and *Kynu* transcripts were increased in these cells from 12 week old R6/2 mice, as found in spleen (Fig. 1d). No difference in the levels of expression of any of the KP enzymes between R6/2 and WT were observed in mice at 4 weeks of age.

### KMO ablation normalises dysregulated expression of KP enzymes in peripheral tissues of R6/2 mice at 12 weeks of age

**Figure 2:**
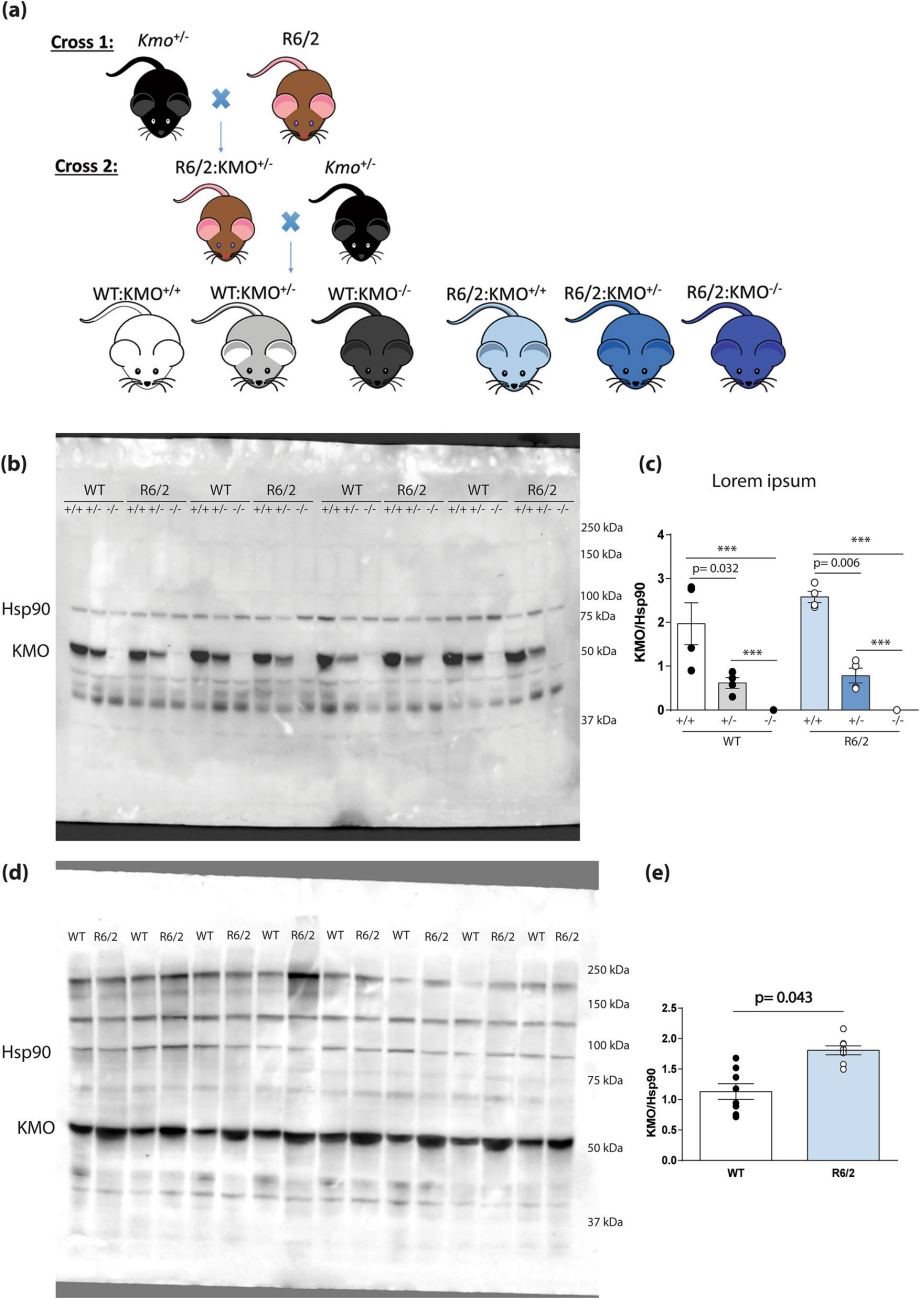
KMO protein levels are increased in R6/2 liver at 12 weeks of age and undetectable after *Kmo* ablation. (**a**) Two intercrosses were performed to generate the six desired genotypes**:** WT:KMO^+/+^ (WT), WT:KMO^+/−^ (*Kmo* heterozygotes), WT:KMO^−/−^ (*Kmo* homozygotes), R6/2:KMO^+/+^ (R6/2), R6/2:KMO^+/−^ (R6/2 *Kmo* heterozygotes) and R6/2:KMO^−/−^ (R6/2 *Kmo* homozygotes) for behavioural and molecular analyses. (**b**) Liver lysates from 12 week old mice for all six genotypes were immunoprobed for KMO by western blot with HSP90 as a loading control (n = 4/genotype) (**c**) Liver KMO levels were reduced in *Kmo* heterozygotes and ablated in *Kmo* knockout homozygotes in both R6/2 and WT mice. (**d**) Liver lysates from 12 week old WT and R6/2 mice were immunoprobed for KMO by western blot with HSP90 as a loading control (n = 8/genotype) (**e**) KMO levels were significantly elevated in R6/2 liver compared to WT littermates. Statistical analyses via one way ANOVA with Bonferroni post hoc correction or Student’s *t*-test; ***p ≤ 0.001. The test statistics, degrees of freedom and *p* values are provided in Supplementary Table S8 online. WT = wild type.

We previously generated *Kmo* knockout mice^23^. In order to transfer the R6/2 transgene onto heterozygous and homozygous *Kmo* knockout backgrounds, together with WT controls, we first bred male R6/2 mice with female *Kmo*^+/−^ heterozygotes to generate R6/2:KMO^+/−^ male mice. These were further bred to *Kmo*^+/−^ female mice to generate the six desired genotypes: WT (WT:KMO^+/+^), *Kmo* heterozygotes (WT:KMO^+/−^), *Kmo* homozygotes (WT:KMO^−/−^), R6/2 (R6/2:KMO^+/+^), R6/2 *Kmo* heterozygotes (R6/2:KMO^+/−^), R6/2 *Kmo* homozygotes (R6/2:KMO^−/−^) (Fig. 2a). We confirmed these KMO genotypes via immunoblotting of liver extracts from the six mouse lines, with a decrease in KMO levels observed in both WT and R6/2 *Kmo* heterozygotes and complete ablation in the respective *Kmo* knockouts (Fig. 2b,c). As KMO levels appeared to be elevated in R6/2 compared to WT littermates, additional liver samples from R6/2 and WT mice were subjected to immunoblotting. KMO levels were found to be ~ 1.5 fold higher in R6/2 liver as compared to WT (Fig. 2d,e), paralleling the elevation in KMO activity previously reported in the brains of R6/2 mice^22^.

**Figure 3:**
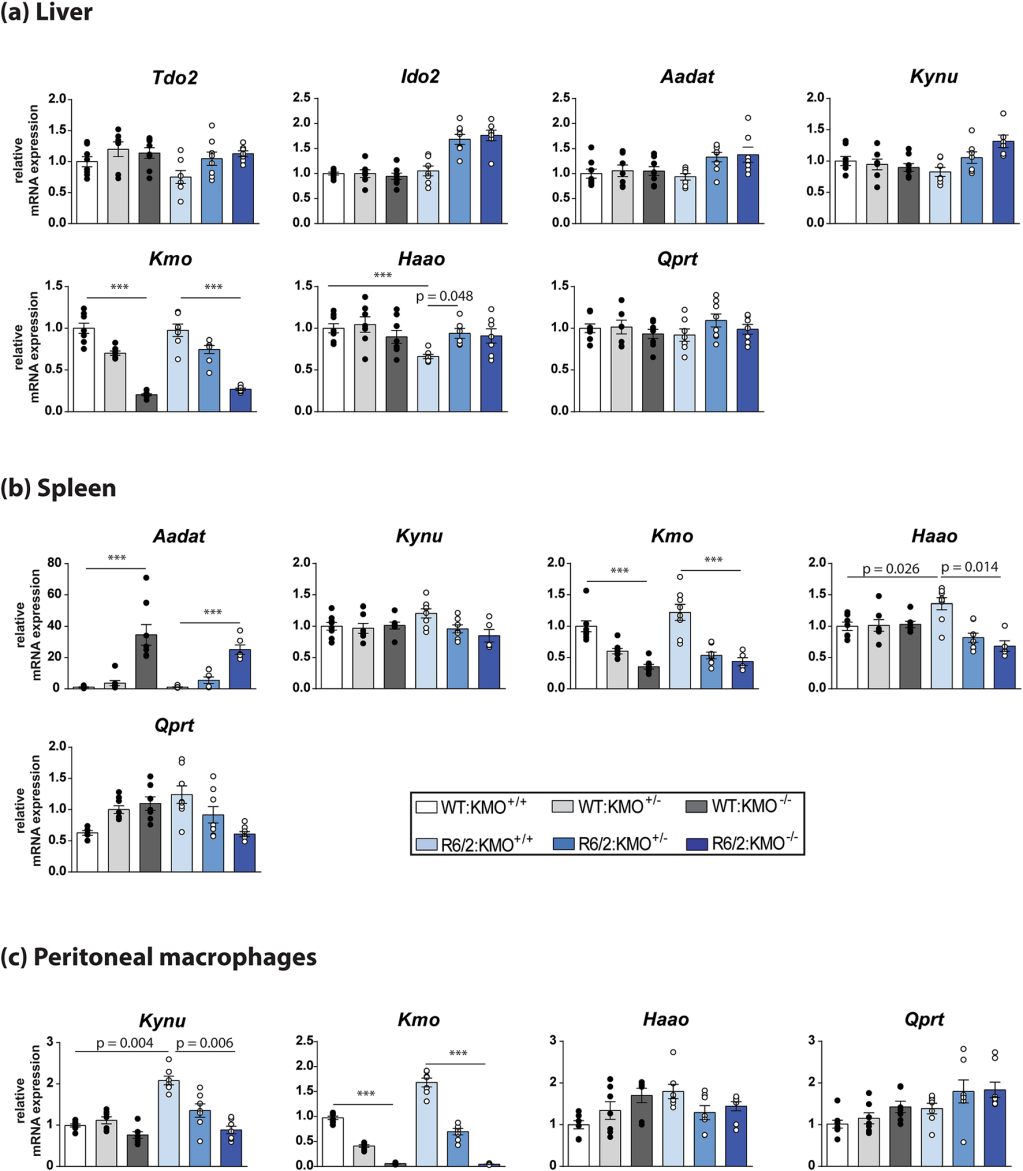
Transcript levels of KP pathway enzymes in response to the ablation of KMO in R6/2 and WT mice at 12 weeks of age. (**a**–**c**) Comparison of the levels of KP enzyme transcripts, as measured by qPCR, between WT and R6/2 mice heterozygous or homozygous knockout for *Kmo* at 12 weeks of age in liver, spleen, and peritoneal macrophages. (**a**) Liver *Kmo* transcript levels were reduced in WT and R6/2 mice heterozygous and homozygous for *Kmo* knockout. Liver transcript levels for *Haao* were restored in R6/2 mice heterozygous for *Kmo*. (**b**) Spleen *Kmo* transcript levels were reduced in both WT and R6/2 mice homozygous and heterozygous for *Kmo* knockout. Spleen transcript levels for *Aadat* were significantly increased in both WT and R6/2 mice homozygous for *Kmo* knockout. Spleen transcript levels for *Haao* were restored in R6/2 mice both homozygous and heterozygous for *Kmo* (**c**) Peritoneal macrophage *Kmo* transcript levels were reduced in WT and R6/2 mice heterozygous and homozygous for *Kmo* knockout. *Kynu* levels were restored in R6/2 mice homozygous for *Kmo*. Statistical analyses via one way ANOVA with Bonferroni post hoc correction; ***p ≤ 0.001, n = 4 per gender/genotype. The test statistic, degrees of freedom and *p* values for the ANOVA are provided in Supplementary Table S9 online. WT:KMO^+/+^  = WT, WT:KMO^+/−^  = *Kmo* heterozygotes, WT:KMO^−/−^  = *Kmo* homozygotes, R6/2:KMO^+/+^  = R6/2, R6/2:KMO^+/−^  = R6/2 *Kmo* heterozygotes, R6/2:KMO^−/−^  = R6/2 *Kmo* homozygotes, WT = wild type.

To investigate whether ablation of KMO had an effect on KP enzyme transcript levels, we measured expression in the liver, spleen and peritoneal macrophages by qPCR in all six genotypes at 12 weeks of age (Fig. 3). *Kmo* transcript levels were decreased in the liver, spleen and peritoneal macrophages, consistent with *Kmo* gene dosage (Fig. 3). Prominent changes in KP enzyme levels in response to the reductions in KMO included normalisation of *Haao* levels in the liver (Fig. 3a) and spleen (Fig. 3b) of R6/2 mice, and *Kynu* levels in R6/2 peritoneal macrophages (Fig. 3c). Notably, *Aadat* levels were significantly increased in spleen tissue upon KMO ablation (38-fold in WT; 20-fold in R6/2), with no apparent effect in the *Kmo* heterozygous knockout state (Fig. 3b).

### KMO ablation in R6/2 mice results in elevated levels of L-KYN, KYNA, 3-HK and AA in the liver and spleen, with a strong reduction in QUIN levels

**Figure 4:**
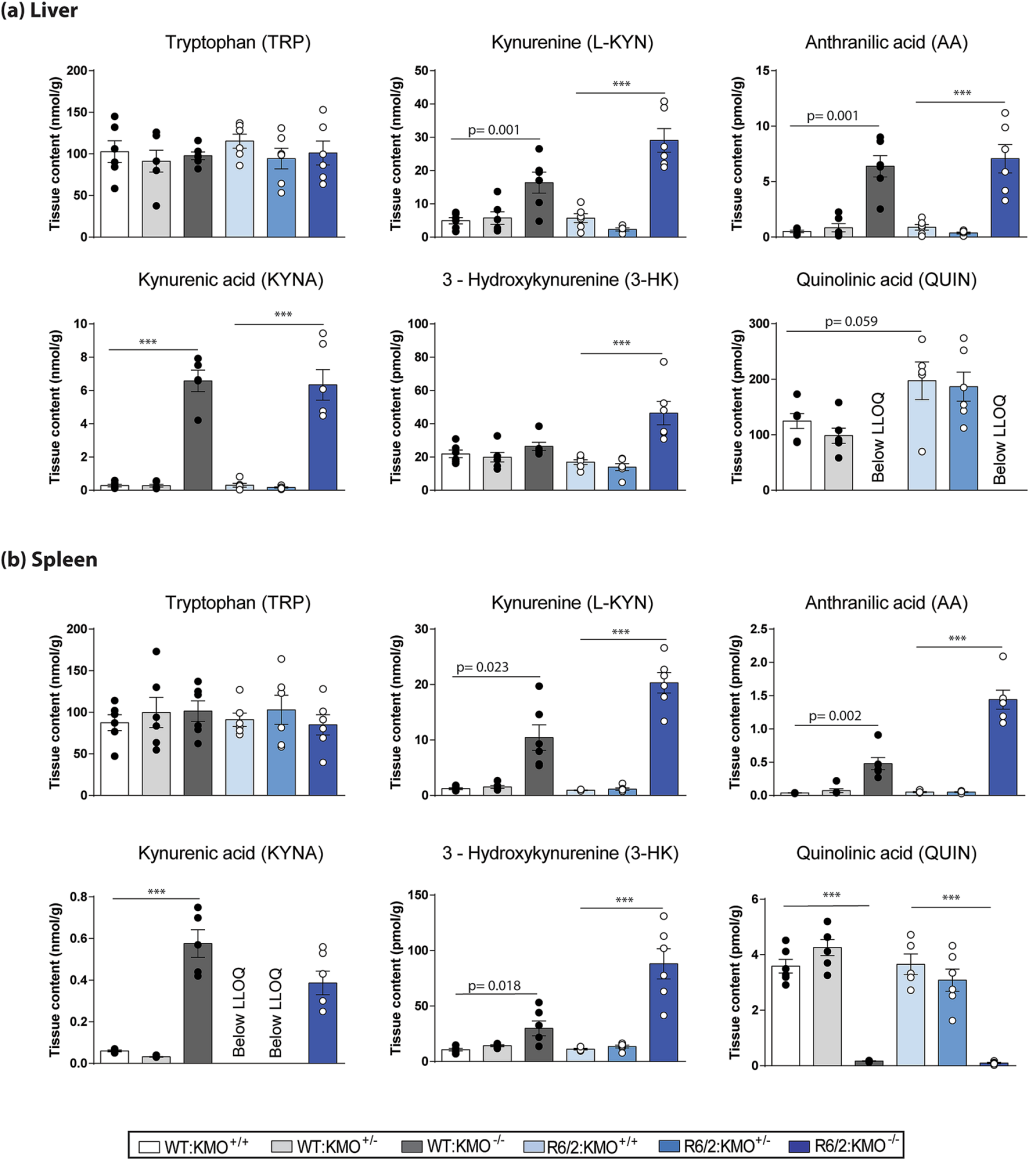
Peripheral metabolites show alteration upon complete KMO ablation only. (**a**–**b**) Liver and spleen metabolites were analysed by HPLC in all six genotypes at 12 weeks of age. (**a**) In the liver, TRP remained unchanged, whereas L-KYN, AA and KYNA were all increased in WT and R6/2 mice homozygous for *Kmo* knockout. 3-HK was increased in R6/2 mice upon KMO ablation. QUIN was significantly increased in R6/2 mice compared to WT with no detectable signal in mice homozygous for *Kmo* knockout. (**b**) In the spleen, TRP remained unchanged, whereas L-KYN, AA, KYNA and 3-HK were all increased in both R6/2 and WT mice homozygous for *Kmo* knockout. QUIN was dramatically decreased in R6/2 and WT mice homozygous for *Kmo* knockout. Statistical analyses via one way ANOVA with Bonferroni post hoc correction; ***p ≤ 0.001, n = 3 per gender/genotype. The test statistic, degrees of freedom and *p* values for the ANOVA are provided in Supplementary Table S10 online. WT:KMO^+/+^  = WT, WT:KMO^+/−^  = *Kmo* heterozygotes, WT:KMO^−/−^  = *Kmo* homozygotes, R6/2:KMO^+/+^  = R6/2, R6/2:KMO^+/−^  = R6/2 *Kmo* heterozygotes, R6/2:KMO^−/−^  = R6/2 *Kmo* homozygotes, WT = wild type.

Due to the altered expression of KP enzymes in the periphery of R6/2 mice, we next analysed KP metabolite levels by HPLC in liver and spleen from all six genotypes deriving from the R6/2 × *Kmo* knockout intercross. In both the liver and spleen, TRP was unchanged in all genotypes (Fig. 4a,b). On the other hand, L-KYN, AA and KYNA levels were all dramatically elevated in both R6/2 and WT mice upon KMO ablation, with no effect observed in *Kmo* heterozygous knockout mice (Fig. 4a,b). Surprisingly, 3-HK levels were elevated in R6/2:KMO^−/−^ mice in the liver, as well as in the spleen of R6/2:KMO^−/−^ and WT:KMO^−/−^ mice (Fig. 4a,b). QUIN levels were significantly increased in the livers of R6/2 mice in comparison to WT littermates, and could not be detected in R6/2:KMO^−/−^ or WT:KMO^−/−^ mice. In spleen, QUIN was significantly reduced in R6/2:KMO^−/−^ and WT:KMO^−/−^ mice (Fig. 4a,b).

### KMO ablation in R6/2 mice results in elevated levels of L-KYN, AA and KYNA and reduced levels of 3-HK and QUIN in the brain

**Figure 5:**
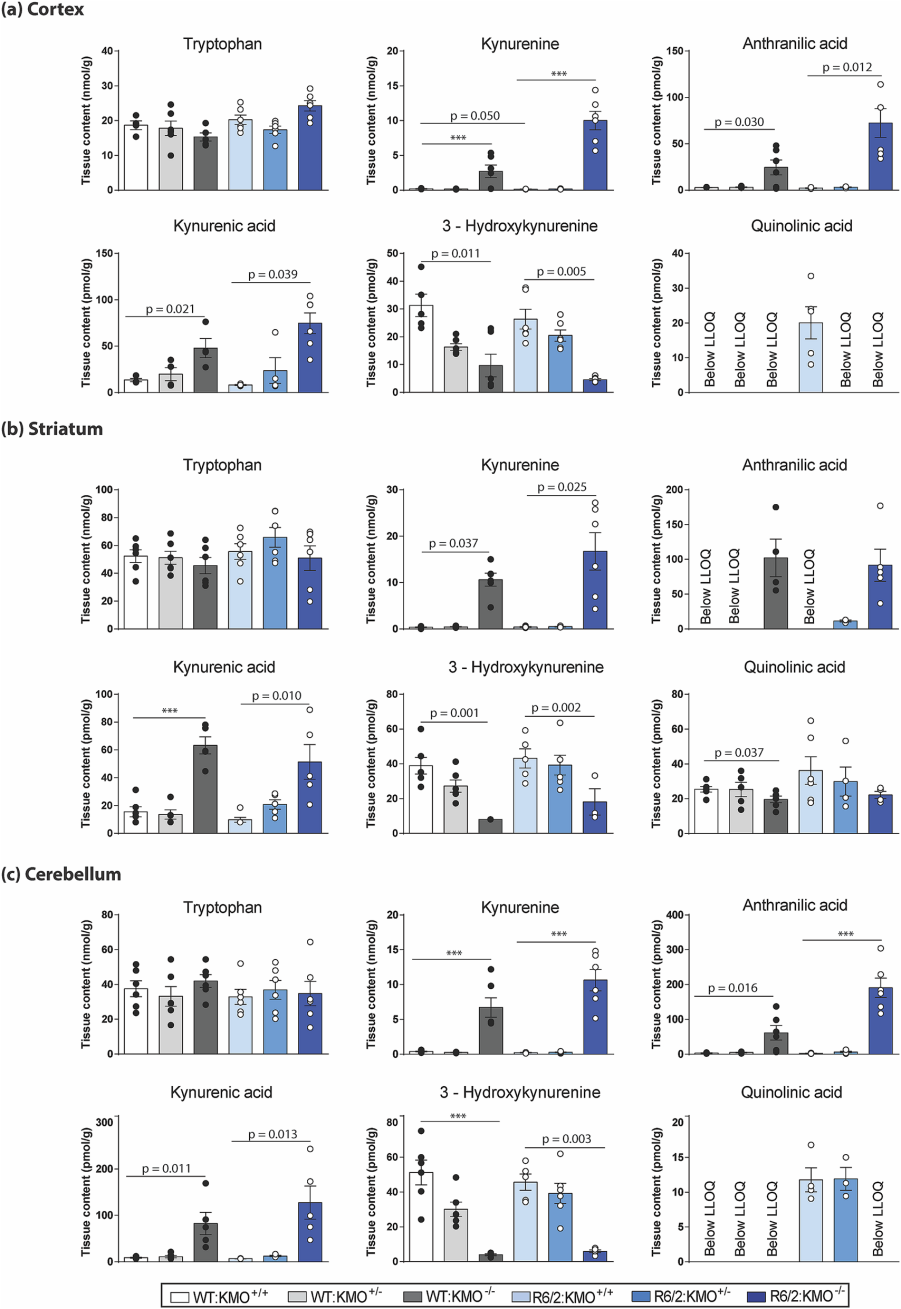
Brain region metabolites show alteration upon complete *Kmo* ablation. (**a**–**c**) Cortical, striatal and cerebellar metabolites were analysed by HPLC in all six genotypes at 12 weeks of age. (**a**) In the cortex, TRP remained unchanged, whereas L-KYN, AA and KYNA were all increased in WT and R6/2 mice homozygous for *Kmo* knockout. 3-HK was decreased in R6/2 mice upon KMO ablation. QUIN did not reach levels of detection except in R6/2 mice, suggesting an increase in levels. (**b**) In the striatum, TRP remained unchanged, whereas L-KYN, AA and KYNA were all increased in WT and R6/2 mice homozygous for *Kmo* knockout. 3-HK was decreased in R6/2 mice upon KMO ablation. QUIN levels were decreased in WT mice homozygous for *Kmo* knockout. (**c**) In the cerebellum, TRP remained unchanged, whereas L-KYN, AA and KYNA were all increased in WT and R6/2 mice homozygous for *Kmo* knockout. 3-HK was decreased in R6/2 mice upon KMO ablation. QUIN did not reach levels of detection except in R6/2 mice and R6/2 heterozygous to *Kmo* knockout. Statistical analyses via one way ANOVA with Bonferroni post hoc correction; ***p < 0.001, n = 3 per gender/genotype, LLOQ—lower limit of quantification. The test statistic, degrees of freedom and *p* values for the ANOVA are provided in Supplementary Table S11 online. WT:KMO^+/+^  = WT, WT:KMO^+/−^  = *Kmo* heterozygotes, WT:KMO^−/−^  = *Kmo* homozygotes, R6/2:KMO^+/+^  = R6/2, R6/2:KMO^+/−^  = R6/2 *Kmo* heterozygotes, R6/2:KMO^−/−^  = R6/2 *Kmo* homozygotes, WT = wild type.

Unlike the KP enzyme transcript levels, KP metabolites could be detected in brain, and therefore these were measured by HPLC from cortex, striatum and cerebellum from all six genotypes of the R6/2 × *Kmo* knockout intercross at 12 weeks of age. In all cases, TRP levels were unchanged (Fig. 5a–c). In all brain regions, L-KYN, AA and KYNA were elevated in both R6/2 and WT upon *Kmo* homozygous knockout (Fig. 5a–c). In contrast to the peripheral tissues, in which 3-HK was elevated, in brain, it was significantly reduced in R6/2 and WT mice homozygous for *Kmo* knockout, as expected (Fig. 5a–c). QUIN levels were very low in all brain regions, and therefore any comparative analyses were difficult to interpret (Fig. 5a–c).

### Altered plasma inflammatory cytokines TNFα, IL1β, IL4, IL5 and IL6 levels were attenuated upon KMO ablation in R6/2 mice

**Figure 6:**
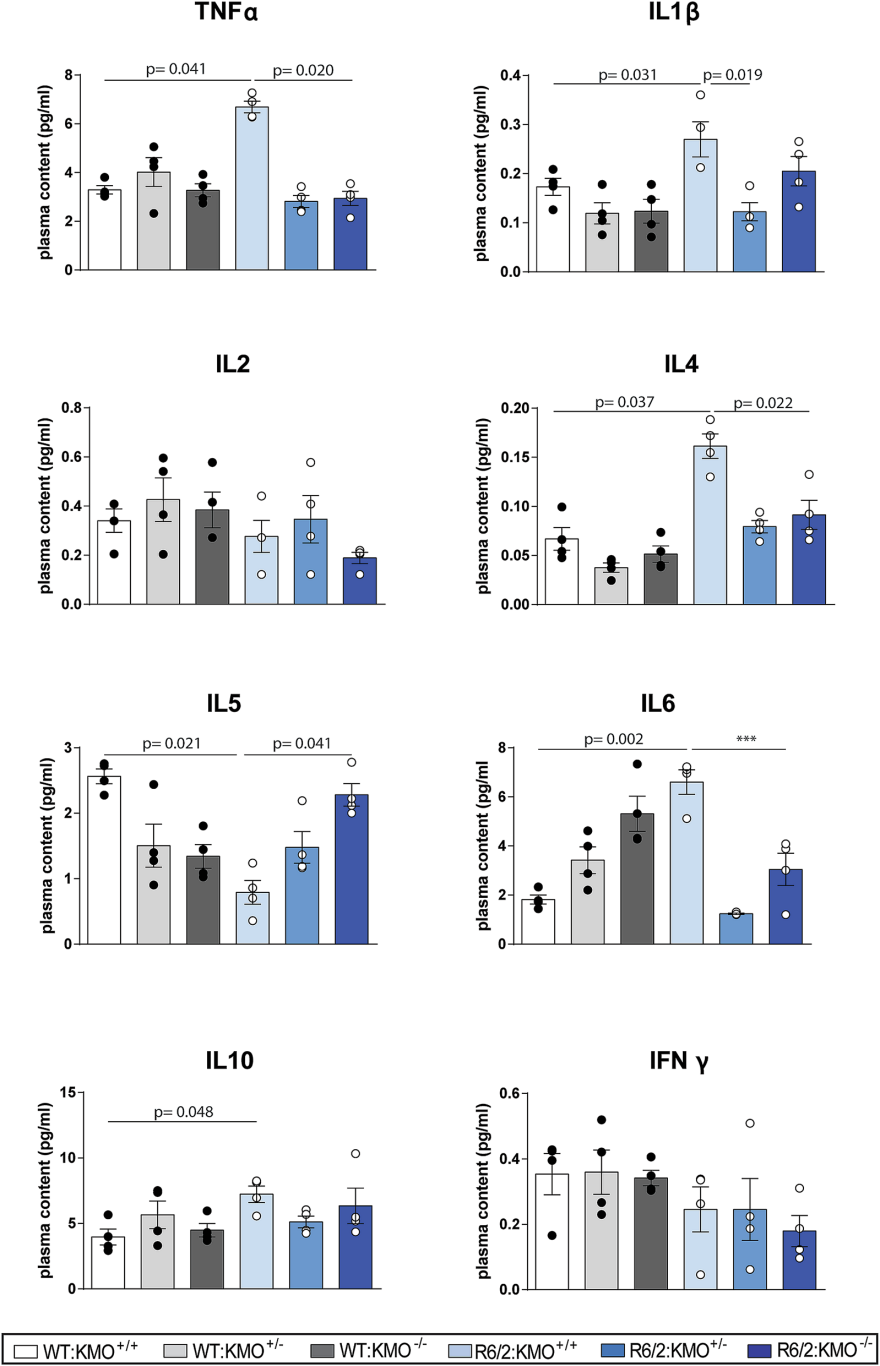
Plasma cytokines TNFα, IL1β, IL4, IL5, IL6 were dysregulated in late stage R6/2 mice and levels rescued upon KMO ablation. Plasma cytokine levels were measured by Mesoscale Discovery technology for all six genotypes at 12 weeks of age. TNFα, IL1β, IL4, IL5, IL6 and IL10 levels were all altered in R6/2 as compared to WT. Both hetero- and homozygosity for *Kmo* resulted in rescue of these levels for all cytokines except IL10, and IL-5 was only rescued upon *Kmo* knockout. Statistical analyses via one-way ANOVA with Bonferroni post hoc correction. The test statistic, degrees of freedom and *p* values for the ANOVA are provided in Supplementary Table S12 online. WT:KMO^+/+^  = WT, WT:KMO^+/−^  = *Kmo* heterozygotes, WT:KMO^−/−^  = *Kmo* homozygotes, R6/2:KMO^+/+^  = R6/2, R6/2:KMO^+/−^  = R6/2 *Kmo* heterozygotes, R6/2:KMO^−/−^  = R6/2 *Kmo* homozygotes, WT = wild type.

Previous studies have shown that pro-inflammatory cytokine levels are increased in the blood and peripheral tissues of mouse models of HD and HD patients, indicative of chronic immune activation during the course of the disease^15,16,24^. These increases occurred in both R6/2 transgenic mice as well as knock-in models^16^. Given that KMO has been shown to influence cytokine levels and that inflammatory cytokines can upregulate the level of transcripts for KMO^19,25^, we used the Mesoscale Discovery platform to measure the plasma levels of TNFα, IL1β, IL2, IL4, IL5, IL6, IL10 and IFNγ in the six genotypes at 12 weeks of age. We found significant increases in R6/2 as compared to WT for TNFα, IL1β, IL4, IL6 and IL10, while IL5 was reduced (Fig. 6). Both hetero- and homozygosity for *Kmo* rescued these levels for all cytokines except IL10; and IL5 was only rescued upon KMO ablation (Fig. 6). There were no changes for IL2 or IFNγ (Fig. 6).

### *Kmo* transcripts visualised in liver Kupffer cells were eliminated in *Kmo* knockout mice

**Figure 7:**
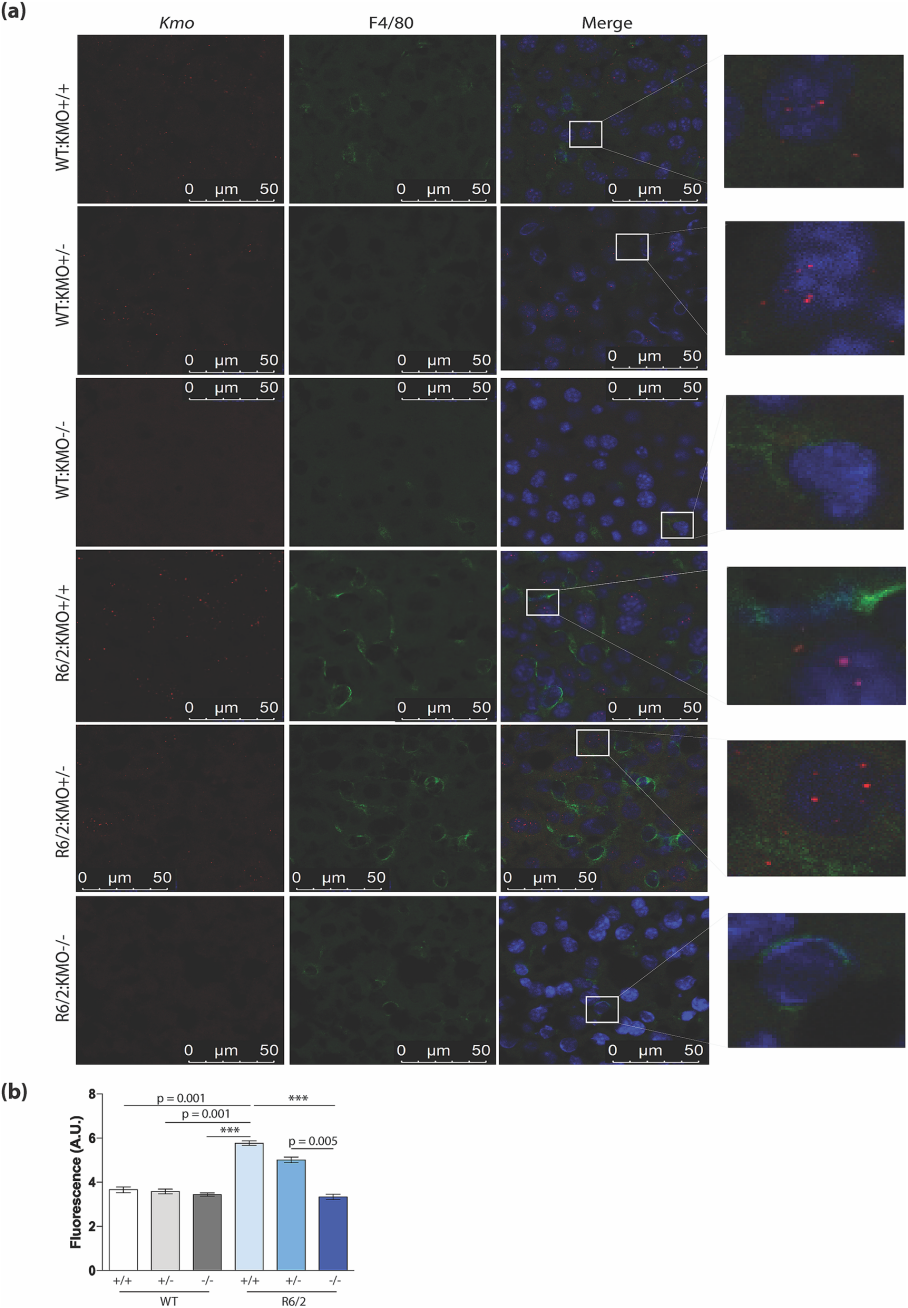
*Kmo* transcripts were visualised in Kupffer cells and eliminated in KMO knockout mice. (**a**) *Kmo* transcript levels in liver were visualised using RNAScope and localised to macrophagic Kupffer cells by co-immunohistochemistry with the F4/80 antibody. The absence of a *Kmo* signal in the *Kmo* knockout sections demonstrated that the *Kmo* probe was specific (3rd and 6th rows). Activation of Kupffer macrophages was more pronounced in liver from R6/2 mice at 12 weeks of age than their WT littermates. Ablation of KMO decreased the F4/80 signal in R6/2 mice to WT levels. The panels on the right are zoomed images of the area on interest in the merged panels. (**b**) Fluorescence intensity for F4/80 signal (arbitrary units) were analysed using image J, based on 30 images per animal. The test statistic, degrees of freedom and *p* values for the ANOVA are provided in Supplementary Table S13 online. ***p < 0.001, n = 3 per genotype. WT:KMO^+/+^  = WT, WT:KMO^+/−^  = *Kmo* heterozygotes, WT:KMO^−/−^  = *Kmo* homozygotes, R6/2:KMO^+/+^  = R6/2, R6/2:KMO^+/−^  = R6/2 *Kmo* heterozygotes, R6/2:KMO^−/−^  = R6/2 *Kmo* homozygotes, WT = wild type.

As we had found that the highest KP enzyme transcripts were in liver (Fig. 1b), we used RNAScope to localize *Kmo* transcripts in this tissue. The *Kmo* knockout tissue therefore acted as a negative control for the specificity of the RNAScope probe (Fig. 7). Given that we had found that ablation of KMO normalised cytokine levels in plasma, the RNAScope was combined with F4/80 immunohistochemistry to identify activated macrophages^26^. We found that *Kmo* transcripts were localised to activated macrophagic Kupffer cells. Interestingly, both visually, and when measuring fluorescence intensity, the activation of these cells was significantly more pronounced in R6/2 liver than in WT at 12 weeks of age (Fig. 7). Ablation of KMO significantly decreased the level of F4/80 staining to WT levels with not apparent changes in R6/2 heterozygous for *Kmo* knockout (Fig. 7). These data were consistent with the decrease in inflammatory plasma cytokines (Fig. 6).

### Behavioural HD phenotypes of R6/2 showed no difference upon KMO ablation

To determine whether KMO ablation had any effect on the course of disease in the R6/2 mice, body weight, grip strength, rotarod performance and locomotor activity were measured. Clasping was not measured as this phenotype is semi-quantitative. Males and females for all six genotypes were assessed. All mice were sacrificed at 13 weeks of age at which point they had reached end-stage disease.

**Figure 8:**
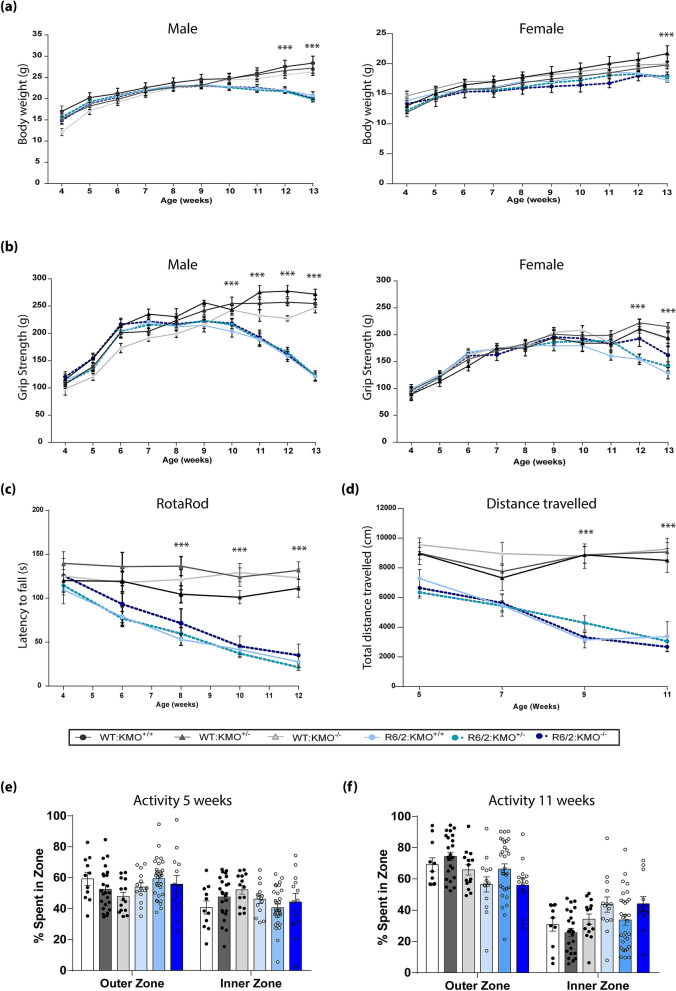
Progression of behavioural and physiological phenotypes of R6/2 mice were not altered upon KMO ablation. (**a**) Body weight measurement of male and female mice for all six genotypes. All R6/2 genotypes failed to gain weight with age as compared to WT littermates, and this was more prominent in males than females. *Kmo* knockout had no effect on body weight. (**b**) Fore- and hind- limb grip strength for male and female mice for all six genotypes. A statistically significant reduction in grip strength occurred in all R6/2 genotypes from 11 and 12 weeks of age for male and female mice respectively. *Kmo* knockout had no effect on grip strength. (**c**) Rotarod performance of male and female mice combined for all six genotypes. Rotarod performance deteriorated with age for all R6/2 genotypes as compared to their WT littermates. *Kmo* knockout had no effect on rotarod performance (**d**) Total distance travelled in the open field for males and females combined for all six genotypes. All R6/2 genotypes developed a general age-related hypoactivity compared to WT littermate mice. *Kmo* knockout had no effect on open field behaviour. (**e**) Time spent in outer and inner zone of the open field arena for both males and females combined for all six genotypes at 5 and 11 weeks of age. R6/2 mice showed no changes in time spent in the outer zone of the arena (thigmotaxis) as compared to their WT littermates irrespectively of *Kmo* knockout. WT:KMO^+/+^ male (n = 11), WT:KMO^+/−^ male (n = 13), WT:KMO^−/−^ male (n = 10), R6/2:KMO^+/+^ male (n = 10), R6/2:KMO^+/−^ male (n = 10), R6/2:KMO^+/−^ male (n = 10), WT:KMO^+/+^ female (n = 12), WT:KMO^+/−^ female (n = 12), WT:KMO^−/−^ female (n = 10), R6/2:KMO^+/+^ female (n = 10), R6/2:KMO^+/−^ female (n = 12), R6/2:KMO^−/−^ female (n = 11). Statistical analysis was one-way ANOVA with Bonferroni post hoc correction, and general linear model. ***p ≤ 0.001, WT:KMO^+/+^  = WT, WT:KMO^+/−^  = *Kmo* heterozygotes, WT:KMO^−/−^  = *Kmo* homozygotes, R6/2:KMO^+/+^  = R6/2, R6/2:KMO^+/−^  = R6/2 *Kmo* heterozygotes, R6/2:KMO^−/−^  = R6/2 *Kmo* homozygotes, WT = wild type.

Body weight was measured weekly from 4 to 13 weeks of age. In all cases, R6/2 mice failed to continue to gain weight relative to WT littermates and the decrease in weight was more pronounced in males than in females. R6/2 × KMO males [F(Age × Genotype)_45,460_ = 3.033, *p* < 0.001] (Fig. 8a). In females the overall interaction between genotype and age was not statistically significant: R6/2 × KMO females [F(Age × Genotype)_45,480_ = 0.683, *p* = 0.943]. However, age and genotype independently had a significant overall effect. R6/2 × KMO females [F(Age)_9,480_ = 41.3421, *p* < 0.001]; R6/2 × KMO females [F(Genotype)_5,480_ = 12.603, *p* < 0.001] (Fig. 8a).

Grip strength was measured at the beginning of every week. Grip strength decreased for R6/2 males compared to WT males: R6/2 × KMO males [F(Age × Genotype)_45,460_ = 9.227, *p* < 0.001] (Fig. 8b). For females the overall interaction between genotype and age was not statistically significant: R6/2 × KMO females [F(Age × Genotype)_4,90_ = 0.554, p = 0.940] (Fig. 8b). However, age and genotype independently had a significant overall effect: R6/2 × KMO females [F(Age)_9,240_ = 114.367, *p* < 0.001] and R6/2 × KMO females [F(Genotype)_5,460_ = 37.217, *p* < 0.001] (Fig. 8b).

To assess changes in balance and co-ordination, mice were tested for their ability to walk on an accelerating rotarod during weeks 4, 6, 8, 10 and 12. There was no difference between the six genotypes at 4 weeks of age [F(Genotype)_5,98_ = 0.674, *p* = 0.644] (Fig. 8c). In all R6/2 genotypes, performance deteriorated with disease progression and there was no effect of the KMO genotype: R6/2 × KMO [F(Age × Genotype)_20,465_ = 2.526, *p* < 0.000] (Fig. 8c).

Exploratory activity in the open field was measured at 5, 7, 9 and 11 weeks of age. The total distance travelled decreased in R6/2 mice with age as compared to WT littermates [F(Age)_3,400_ = 12.707, *p* < 0.000] (Fig. 8d) and this was not modified by KMO genotype [F(Age × Genotype)_15,400_ = 3.315 , *p* < 0.001] (Fig. 8d). Diminished exploration was not accompanied by anxiety in any of the genotypes. R6/2 compared to WT genotypes did not show altered thigmotaxis over the course of the trial R6/2 × KMO [F(Age × Genotype)_15,400_ = 1.445, *p* = 0.123] (Fig. 8e,f and Supplementary Figure S1 online).

## Discussion

There is mounting evidence for KMO inhibition in the treatment of a wide range of brain disorders^11,12,20^, predominantly aimed at reducing the neurotoxic metabolites 3-HK and QUIN, while increasing levels of neuroprotective KYNA. Based on previous evidence, here we have crossed the R6/2 transgene onto a KMO knockout background to investigate the effects of genetic KMO ablation on KP metabolites and enzymes, as well as the potential phenotypic consequences. The absence of KMO had no adverse effects on the onset and progression of the R6/2 phenotype. We found that tissue KP metabolite concentration was modulated as expected, indicating that no compensatory mechanisms to counteract constitutive ablation had occurred. Therefore, neurotoxic metabolite levels were largely decreased, and neuroprotective levels increased in both the brain and periphery. The levels of several KP enzyme transcripts, distal to KMO, that were dysregulated in R6/2 mice were restored in WT animals. We found KMO ablation decreased liver inflammation and restored the plasma levels of several proinflammatory cytokines in R6/2 mice, however, despite these beneficial effects, we found no improvement in weight loss or behavioural assessments.

The neurotoxic metabolites of the KP have been shown to be elevated in HD patients in the neostriatum and cortex, i.e. two brain areas which suffer pronounced neuronal loss in HD, while neuroprotective KYNA was found to be significantly reduced in the striatum^27^. Furthermore, studies in HD mouse models have shown 3-HK and QUIN metabolite levels, as well as KMO activity, to be increased in the striatum and cortex prior to the emergence of overt neuronal loss^28^. Consistent with these findings we found QUIN to be increased in the cortex and cerebellum of R6/2 compared to WT littermates (Fig. 5). In keeping with the previous analysis of the KMO knockout mouse^23^, we found L-KYN, AA and KYNA to be elevated in the brains of R6/2 and WT mice lacking KMO, whereas metabolites distal to KMO were largely eliminated. The same pattern was observed in liver and spleen, although notably the metabolites in the periphery were present at much higher levels. Unexpectedly, in both liver and spleen, 3-HK, the metabolite directly catalysed by KMO, was elevated via an unknown mechanism (Figs. 1a, 4). This is consistent with our past observation that appreciable levels of 3-HK are found in the liver and plasma of KMO knockout mice in a WT background, while being essentially eliminated in brain tissue^23^.

Surprisingly, we were unable to detect any of the KP enzyme transcript levels in brain. This is consistent with a previous report of very low *Ido* and *Kmo* levels in rat brain^29^. In contrast, all of the KP enzymes were expressed at relatively high levels in liver. This was also true for the distal KP enzymes in spleen and peritoneal macrophages, with the proximal enzymes, *Tdo2*, *Ido2* and *Aadat* being expressed at low levels or undetectable. There were no differences in KP enzyme levels in the peripheral tissues of pre-symptomatic R6/2 mice at 4 weeks of age as compared to WT, and whilst several of the enzymes were dysregulated at 12 weeks, this had no effect on metabolites. Upon KMO ablation, the levels of these dysregulated transcripts were normalised. Interestingly, in the absence of KMO, the level of *Aadat* in the spleen was increased 25 and 48 fold in WT and R6/2 respectively, and this upregulation of *Aadat* is most likely responsible for the spleen-specific increase in KYNA. These dramatic changes in *Aadat* levels may reflect an increased requirement for the activity of the enzyme it encodes (AADAT/kynurenine aminotransferase II) due to excess levels of L-KYN arising from *Kmo* ablation.

It is well documented that chronic inflammation is altered in the periphery of HD patients and mouse models^14–16,30^. KP metabolism itself is modulated in conditions such as infection and stress and several studies suggest that when the immune system is activated, cytokines stimulate activity of KP enzymes both in periphery and CNS^19,31^. Although no previous studies have linked immunomodulation in HD and the KP, several studies have reported that immune rescue in HD mouse models has therapeutic benefits^32,33^, thus immunosuppression via KMO inhibition may provide a potential therapeutic strategy for HD. In view of this, we investigated whether KMO inhibition had an effect on peripheral inflammation, as several KP metabolites can readily cross the BBB barrier, and neurotoxic cytokines (e.g. IL1β, IL6) are dysregulated in periphery during HD^19,30^. Interestingly, we found dysregulation of the inflammatory cytokines TNFα, IL1β, IL4, IL5 and IL6 in plasma to be rescued upon KMO ablation in R6/2 mice. Furthermore, we found that the elevated IL10 levels, observed in R6/2 mice, were not rescued upon KMO knockout and since this cytokine has anti-inflammatory activity, it may further protect against chronic inflammation in R6/2 mice. That inflammation might be linked to bioactive TRP metabolites was predicted in a recent review for a wide range of neurodegenerative diseases^34^. Furthermore, exploring the NLRP3 inflammasome, which plays a crucial role in both acute and chronic inflammation, in this context could provide some novel insight into the inflammatory phenotypes we observed^35^. Aggregated proteins sensed by the NLRP3 inflammasome have previously been shown to activate caspase-1 and thereby upregulate levels of IL1β and IL18^35^. Following on from this, it has also been shown that L-KYN can act as an antagonist for AHR nuclear receptors in vitro, which thereby may inhibit the NLPR3 inflammasome^36^. These could thus all potentially contribute to the modulation of peripheral inflammation we observed.

Tissue-specific macrophages are highly specialised phagocytic cells that play a critical role in inflammation^37^. Of these, Kupffer cells are present in the liver and play an important role clearing debris from the blood and regenerating damaged liver tissue^38^. When activated these cells can release an array of growth factors, inflammatory mediators, and oxygen species^39^. Activated macrophages are able to produce a pro-inflammatory response triggering cytokines such as IL6, IL1, IL12 and TNFα and anti-inflammatory IL10^40,41^. We found an increase in activated Kupffer cells in R6/2 mice compared to WT littermates indicating an increase in inflammation. Interestingly, R6/2 mice with ablated KMO show a diminished Kupffer cell profile. The effect of KMO ablation on neuropathology was not studied as we do not observe overt neuronal loss or neuroinflammation in the R6/2 brain at late-stage disease.

KYNA and L-KYN have previously been reported to attenuate inflammation. KYNA can alleviate inflammatory conditions by several means, including restricting TNF production of macrophages via G protein-coupled receptor GPR35 activation^42^. KYNA also decreases IL4 secretion from T-cell receptor stimulated variant natural killer-like T cells and IL23 from LPS-induced dendritic cells^43^. L-KYN regulates inflammation largely through its function as a ligand for the aryl hydrocarbon receptor (AHR), a transcription factor that controls local and systemic immune responses. Several studies have suggested that KYN/AHR signalling is critical for the maintenance of long-term immunosuppression^44^. Given the anti-inflammatory effects of L-KYN and KYNA, elevated levels of these metabolites, due to the upregulation of *Aadat* in spleen in KMO ablated R6/2 mice (Figs. 3b, 4, 5), could explain the decreased inflammatory responses observed.

Herein, we show that in vivo genetic ablation of KMO in the R6/2 HD mouse model eliminated neurotoxic, and increased neuroprotective, metabolites in both the brain and periphery. Furthermore, KMO ablation was able to rescue and normalise TNFα IL1β, IL4, IL5 and IL6 in R6/2 plasma and decrease inflammation in the liver. However, despite these beneficial consequences, a longitudinal assessment of HD-associated phenotypes from four weeks of age revealed that KMO ablation had no effect on the extent or progression of weight loss, grip strength impairment, rotarod performance or locomotor activity. Similarly, although beneficial effects of treating R6/2 mice with small molecule inhibitors of KMO have been reported^11,12^, these studies also did not show notable improvements in behavioural measures. Pharmacological inhibition has primarily acted via the periphery and therefore we aimed to investigate whether global *Kmo* knockout would elicit a more profound effect on the KP and therefore more strongly manipulate key neuro-metabolites which may be contributing to HD phenotypes. Taken together, our work and that of others indicate that whilst KMO inhibition can modulate selected phenotypes, this therapeutic approach is unlikely to act as a major means of providing disease modification for HD. More broadly, this work suggests that dampening of the peripheral immune response in HD may also not be a viable therapeutic intervention. Nonetheless, KMO inhibition still holds promise for treatment of disorders such as Alzheimer’s and Parkinson’s diseases, where amelioration of disease phenotypes has been observed in fruit fly and mouse models of these disorders^11,13^. Furthermore, as Alzheimer’s and Parkinson’s have a much more pronounced state of neuroinflammation as well as a profound induction of the KP via pro-inflammatory cytokines^7^ perhaps in this instance rescuing these mediators will alleviate the symptoms more dramatically.

## Materials and methods

### Mouse breeding and maintenance

The research conducted herein complied with the ARRIVE guidelines (http://www.nc3rs.org.uk/page.asp?id=1357). All procedures were performed in accordance with the Animals (Scientific Procedures) Act 1986 and approved by the University College London Ethical Review Committee. R6/2 mice^45^ were bred by backcrossing R6/2 males to C57BL/6JOlaHsd × CBA/CaOlaHsd F1 females (B6CBAF1/OlaHsd, Envigo, Netherlands). *Kmo* knockout mice^23^ were bred by backcrossing to C57BL/6 J (Charles River). All mice were group housed dependent on gender, but genotypes were mixed within cages. All animals had unlimited access to food (Envigo) and water, were provided with environmental enrichment^46^ which included chew sticks and play tubes, and were maintained under a 12 h light/dark cycle. The animal facility was barrier-maintained and quarterly non-sacrificial FELASA screens found no evidence of pathogens. Mice were euthanized via cervical dislocation at 4 and 12 weeks of age and tail samples were collected for re-genotyping.

### DNA extraction, genotyping and CAG repeat sizing

Ear biopsies were collected and DNA was extracted as previously described^47^. Genotyping was performed by polymerase chain reaction (PCR) of ear or tail-tip DNA and all primers were from Invitrogen. For R6/2, a 10 μL reaction contained 50–100 ng DNA, 1 × Dream Taq Hot Start Green PCR Master Mix (Thermo Fisher Scientific), 1 μM forward primer [5′-CGCAGGCTAGGGCTGTCAATCATGCT-3′], 1 μM reverse primer [5′-TCATCAGCTTTTCCAGGGTCGCCAT-3′], 1 μM forward internal control primer [5′-AGCCCTACACTAGTGTGTGTTACACA-3′], 1 μM reverse internal control primer [5′-CTTGTTGAGAACAAACTCCTGCAGCT-3′]. PCR was carried out in T100 Thermal Cycler (Bio-Rad) using the following cycling conditions: 3 min at 95 °C, 34 × (30 s at 95 °C, 30 s at 60 °C and 1 min at 72 °C), 15 min at 72 °C. For *Kmo* genotyping, a 20 μL reaction contained 50–100 ng DNA, GoTaq Flexi Buffer (Invitrogen), 25 mM MgCl_2_, 2 mM dNTPs (2′ di-deoxy triphosphate, Invitrogen), 1 μM forward primer [5′-AAGCAGAGAAAACTTAAACAAGGAC-3′], 1 μM reverse primer [5′-TTCACCACACAGCTTCCTAACT-3′], 1 μM forward *Kmo* knockout primer [5′-CCTCGTGCTTTACGGTATCGCCGCTC-3′], 1 μM reverse *Kmo* knockout primer [5′-ATGCCTGCAACAACAATCAA-3′]. PCR was carried out in T100 Thermal Cycler (Bio-Rad) using the following cycling conditions: 3 min at 94 °C, 35 × (30 s at 95 °C, 30 s at 60 °C, 60 s at 72 °C), 15 min at 72 °C. The products were separated on a 2% agarose gel at 125 V for 20 min which were then visualised using Image Lab 5.2.1 in Gel Doc XR^+^ (Bio-Rad). For all R6/2 mice, CAG repeat sizing was performed: a 25 μL reaction contained 25—50 ng DNA, 0.2 μM FAM-labelled forward primer [5′- CCTTCGAGTCCCTCAAGTCCTT-3′], 0.2 μM reverse primer [5′-CGGCTGAGGCAGCAGCGGCTGT-3′], AmpliTaq Gold 360 Master Mix (Thermo Fisher Scientific), GC enhancer (Thermo Fisher Scientific). PCR amplification was carried out in T100 Thermal Cycler with following cycling conditions: 10 min at 95 °C, 30 × (30 s at 95 °C, 30 s at 58 °C and 90 s at 72 °C), 7 min at 72 °C. The mean CAG repeat size for all R6/2:KMO^+/+^ mice was 190.55 ± 1.29 (SD), for all R6/2:KMO^+/−^ was 184.24 ± 1.42 (SD) and for all R6/2:KMO^−/−^ mice was 188.24 ± 1.62 (SD).

### Plasma, cell and tissue sample collection

#### Tissue sample collection

Following euthanasia, liver, spleen and brain regions were dissected, flash-frozen in liquid nitrogen and stored at − 80 °C until use.

#### Peritoneal macrophage collection

Following cervical dislocation, 5 mL of ice-cold DMEM medium (Thermo Fisher Scientific) was injected into the mouse abdominal cavity using a 26 gauge needle. After 1–2 min of gentle massage of the abdomen, cells were recovered using a P1000 pipette through a small incision, collected into a 15 mL falcon tube and put on ice. Cells were then centrifuged at 300 × *g* for 5 min at 4 °C. After removing the supernatant, the cell pellet was resuspended in 5 mL 37 °C DMEM medium and incubated at 37 °C for 2 h. The cells were washed twice with PBS, centrifuged at 400 × *g* for 10 min at 4 °C. The purpose of incubation and washing was to remove the non-adherent neutrophils. The cell pellet was immediately frozen on dry ice and stored at − 80 °C until use.

#### Blood/plasma collection

Blood was taken via tail vein puncture post cervical dislocation and collected into EDTA tubes. Blood samples were spun at 2000 × *g* for 5 min and the upper plasma layer removed.

### RNA extraction and real‐time quantitative PCR

Total RNA was extracted using the RNeasy mini kit according to the manufacturer’s instructions, which includes a DNase I treatment to remove genomic DNA (Qiagen). RNA was quantified using a Nanodrop-1000 spectrophotometer (Thermo Fisher Scientific) and stored at − 80 °C. Using the MMLV Superscript reverse transcriptase (Invitrogen) and random hexamers (Operon), reverse transcription (RT) was performed as described previously^48^. Taqman quantitative real-time PCR (qPCR) was performed on the using the CFX96 C1000 real-time thermal cycler (Bio-Rad). The levels of gene expression were determined by normalizing to housekeeping genes, and the relative levels of the transcripts were determined by using the 2^*−ΔΔCt*^ method^48,49^. The expression levels of a ‘gene of interest’ was normalised to the geometric mean of endogenous housekeeping genes. The reference genes were selected from a panel based on their stability in R6/2 and WT samples using the Genorm kit (Primer Design) and were *Hprt*, *Canx,* and *Eif4a2* for liver and *Hprt*, *B2m* and *Atp5b for spleen.* We did not have sufficient RNA to determine the stability of a panel of reference genes for the peripheral macrophages and therefore, these reference genes were based on previous data^16^. Primer sequences and probes used are summarized in Supplementary Table S5 online.

### Protein extraction, SDS PAGE and immunoblotting

Protein extractions, SDS PAGE and immunoblotting were carried out as previously described by Landles et al.^50^, and detailed below. Frozen mouse liver was homogenized (Polytron PT1200E) in ice-cold IGEPAL RIPA buffer [150 mM NaCl, 50 mM Tris–HCl pH 8.0, 1% IGEPAL, 0.5% sodium deoxycholate, 0.1% SDS] with 1 mM PMSF, 1 mM DTT and cOmplete protease inhibitor cocktail tablets (Roche). Protein concentration was determined using the Pierce BCA protein assay kit (Thermo Fisher Scientific). For immunoblotting, 20–40 μg of total protein in Laemmli loading buffer was denatured at 95 °C for 5 min, separated by 12- Criterion TGX Stain-free SDS-PAGE (Bio-Rad) and blotted onto 0.45 μm nitrocellulose membrane (Bio-Rad) by submerged transfer apparatus (Bio-Rad) in [25 mM Tris base, 192 mM glycine, 20%, v/v, methanol]. Membranes were blocked for 1 h at room temperature in 3% non-fat dried milk in PBST [PBS, 0.1% Tween-20], and then incubated overnight with gentle agitation at 4 °C.

Primary antibodies (in PBST with 1% non-fat dried milk) against KMO and loading control HSP90 (Supplementary Table S6 online) were incubated overnight at 4 °C in PBST. For chemiluminescent detection, blots were washed three times in PBST for 5 min, probed with HRP-linked secondary antibodies (in PBST with 0.5% non-fat dried milk) for 1 h at room temperature and washed three times in PBST. Protein was detected by chemiluminescence (Clarity, Bio-Rad) according to the manufacturer's instructions. The signals were captured on a ChemiDoc Touch Imaging System (Bio-Rad).

### High performance liquid chromatography

Concentrations of KP metabolites were determined by HPLC with tandem mass spectrometry (MS/MS) detection as described previously in Beaumont et al.^12^. Tissue extracts samples were prepared by sonication in acetonitrile and centrifugation. L-KYN, KYNA, 3-HK, AA and TRP analysis involved mixing an aliqout of each sample with the internal standards D4-L-KYN, D_5_-KYNA, ^13^C_6_-3-HK, D4-AA and D5-TRP. A separate analytical run was performed for analysis of QUIN, with samples prepared by mixing with the internal standard D_3_-QUIN. The same procedures using blank tissue extracts were employed for the calibrators, QC’s and blanks.

### Mesoscale discovery analysis of cytokine levels in plasma

Cytokine (IL1β, IL2, IL4, IL5 IL6, IL10, IFNγ and TNFα) levels in plasma were quantified using Mesoscale Discovery (MSD) assays in accordance with the manufacturer’s instructions. All reagents were provided as a kit unless stated elsewhere. For greater uniformity, the plate was pre-washed three times using 0.05% PBST [PBS, 0.05% Tween-20]. Calibration was prepared by making six fourfold serial dilutions of Calibrator 1. Plasma samples (in triplicates) were diluted 2.5-fold on the plate with Diluent 41. The plate was sealed and incubated overnight at 4 °C. On the next day, after three washings with 0.05% PBST, the antibody solution containing anti-mouse IFNγ, IL1β, IL2, IL4, IL5, IL6, IL10, TNFα antibodies was added to the plate. Following 2 h incubation with shaking at room temperature, the plate was washed three times by 0.05% PBST, the read-buffer was added and the plate was analysed on a SECTOR 2400 instrument (MSD).

### RNAScope

Liver samples were fresh frozen in OCT (CellPath Ltd) on dry ice and cut at 15 μm on a cryostat and mounted onto slides. Samples were then prepared according to the RNAScope Multiplex Fluorescent v2 Assay protocol (Advanced Cell Diagnostics). Sections were dehydrated in increasing percentages of ethanol and incubated overnight at 100% ETOH at − 20 °C. Hybridization was conducted using RNAScope Multiplex Fluorescent kit v2 (Advanced Cell Diagnostics, Hayward CA). The DNA probe specific for mouse *Kmo* (ACBBio) was incubated for 2 h at 40 °C in a hybridization oven (HybEZ Oven, Advanced Cell Diagnostics). Following RNAScope, samples were stained using the IHC protocol.

### Immunohistochemistry

Prior to immunostaining, non-specific binding was blocked for 1 h using 10% normal goat serum (Sigma) in PBST [0.5% tritonX-100 (Sigma) in PBS]. F4/80 primary antibody (Supplementary Table S6 online) was applied in fresh blocking solution overnight at 4 °C. Sections were washed three times in PBS before application of the biotinylated secondary antibody (Supplementary Table S6 online) for 2 h. Sections were washed three times in PBS followed by DAPI application following the RNAscope Multiplex Fluorescent v2 Assay protocol.

Histological images were obtained using the Multiphoton Microscope Leica TCS SP8 MP and images were recorded using Leica LAS X and analysed with ImageJ software (National Institutes of Health, USA). To calculate the mean fluorescence intensity, TIFF images were imported to ImageJ/Fiji software and threshold function applied in order to separate the signal from the background and the mean signal intensity was measured by the “measure” function^51^. The mean intensity of the background was obtained by averaging the values of negative control images that had been treated with secondary antibody only. The fluorescence intensity level value was calculated by dividing the mean signal intensity above the background for 30 images per mouse for 3 mice per genotype.

### Behavioural and phenotype assessment

For behavioural testing, animals of mixed genotypes were assessed blinded one cage at a time, and in the same order on each trial day.

#### Body weight

Body weight was monitored weekly, prior to grip strength and rotarod assessment, and recorded to the nearest 0.1 g.

#### Grip strength

Fore- and hind-limb grip strength was measured using a grip strength meter (Bioseb) weekly from 4 to 12 weeks of age. Mice were held by the base of the tail and allowed to grip the wire, they were gently pulled horizontally away from the apparatus until the wire was released and the maximum tension (g) was recorded. The mice were tested five times and the average performance of the best three trials was used^52^. The apparatus was thoroughly cleaned with 70% industrial methylated spirit between trials.

#### Rotarod performance

Mice were tested on a standard Ugo Basile 7650 rotarod (Linton Instrumentation, UK), with the modification of a smooth rubber coating over the rotating rod as previously described^52^. At the start of each trial, mice were acclimatized to the apparatus by being placed on the rotating rod at 4 rpm for 20 s. Following acclimatization, the rod progressively accelerated from 4 to 40 rpm over a period of 5 min. Latency (s) for mice to fall from the rod was recorded. For the first test session at 4 weeks, rotarod performance was tested for three trials per day for four consecutive days. For all later sessions, performance was tested for three trials per day for three consecutive days. In both cases, the data from the last two days was used for the analysis. The whole apparatus was thoroughly cleaned with 70% industrial methylated spirit (IMS) between trials.

#### Open field

Open field behaviour was assessed as previously described^53^, and detailed below. Locomotor activity was tested in an open field arena at 5, 7, 9 and 11 weeks of age. Mice were individually placed into plain, featureless white arenas (50 × 50 × 50 cm, Engineering & Design Plastics Ltd., Cambridge, UK) for 30 min. Behaviour was videotaped via a camera positioned above the apparatus. Activity (distance moved, cm) was later tracked and analysed using EthoVision 11.5 XT software (Noldus, Netherlands). Arenas were thoroughly cleaned with 70% industrial methylated spirits between trials.

### Statistical analyses

All data were screened for statistical outliers using ROUT test for behaviour and Grubbs’ Test for molecular analyses (GraphPad Software, California, USA) and outliers were excluded. Statistical analysis was performed with SPSS (v26) (IMB, Portsmouth, UK) using two-tailed Student’s *t*-test, one‐way ANOVA, two‐way ANOVA or GLM ANOVA, with Bonferroni post hoc tests as indicated. Graphs were prepared using Prism Ver. 6 (GraphPad Software, California, USA). *p*-values less than 0.05 were considered statistically significant.

## Supplementary Information

Supplementary information.

## Data Availability

The datasets generated during and/or analysed during the current study are available from the corresponding author on reasonable request.
