# Supplementary material for: Ablation of kynurenine 3-monooxygenase rescues plasma inflammatory cytokine levels in the R6/2 mouse model of Huntington’s disease
Source: Sci Rep. 2021 Mar 9;11:5484. doi: 10.1038/s41598-021-84858-7 (PMC7943810; doi:10.1038/s41598-021-84858-7)
Supplement: Supplementary file 1 — Supplementary information. [file 41598_2021_84858_MOESM1_ESM.pdf]

Supplementary information

Ablation of kynurenine-3-monooxygenase rescues plasma inflammatory cytokine levels in the R6/2 mouse model of Huntington's disease

Marie Katrin Bondulich[1], Yilan Fan[1], Jina Song[1], Flaviano Giorgini[2], and Gillian P Bates[1]

[1] Huntington's Disease Centre, Department of Neurodegenerative Disease and UK Dementia Research Institute at UCL, Queen Square Institute of Neurology, UCL, Queen Square, WC1N 3BG, UK

[2] Department of Genetics and Genome Biology, University of Leicester, Leicester, UK

Corresponding author: fg36@leicester.ac.uk

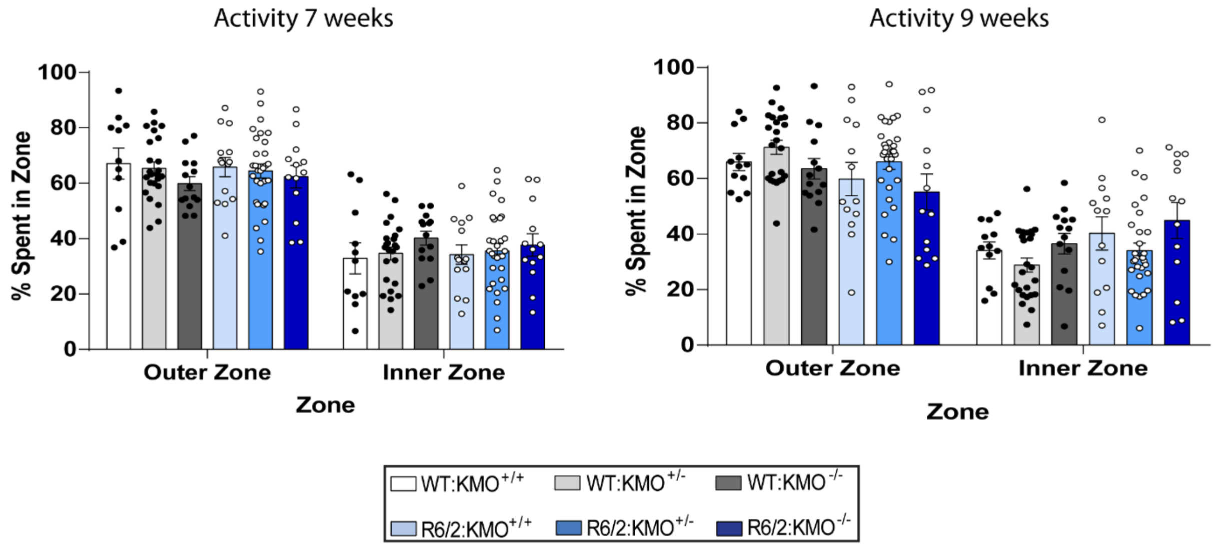

Supplementary Figure S1. **Time spent in outer/inner zone at seven and nine weeks of age.** Seven and nine week old data for time spent in outer and inner zone for both males and females combined for all six genotypes. R6/2 mice showed no changes in time spent in the outer zone of the arena (thigmotaxis) as compared to their wildtype littermates irrespectively of *Kmo* knockout status. Statistical analysis was one way ANOVA with Bonferroni *post hoc* correction; WT:KMO$^{+/+}$ male (n = 11), WT:KMO$^{+/-}$ male (n = 13), WT:KMO$^{-/-}$ male (n = 10), R6/2:KMO$^{+/+}$ male (n = 10), R6/2:KMO$^{+/-}$ male (n = 10), R6/2:KMO$^{+/-}$ male (n = 10), WT:KMO$^{+/+}$ female (n = 12), WT:KMO$^{+/-}$ female (n = 12), WT:KMO$^{-/-}$ female (n = 10), R6/2:KMO$^{+/+}$ female (n = 10), R6/2:KMO$^{+/-}$ female (n = 12), R6/2:KMO$^{-/-}$ female (n = 11). Statistical analysis was one-way ANOVA with Bonferroni *post hoc* correction, and general linear model. ***p ≤ 0.001, WT:KMO$^{+/+}$ = WT, WT:KMO$^{+/-}$ = *Kmo* heterozygotes, WT:KMO$^{-/-}$ = *Kmo* homozygotes, R6/2:KMO$^{+/+}$ = R6/2, R6/2:KMO$^{+/-}$ = R6/2 *Kmo* heterozygotes, R6/2:KMO$^{-/-}$ = R6/2 *Kmo* homozygotes, WT = wild type

Supplementary Table S1. ***Mean qPCR threshold (C_t) values of reference and target genes in brain from R6/2 and wild type mice.***

Cortex	WT		R6/2	
	4week	12week	4week	12week
Tdo2	37.94 (0.97)	38.52 (0.63)	37.69 (0.69)	37.93 (0.53)
Ido2	37.12 (0.89)	38.23 (0.80)	37.20 (1.10)	37.45 (0.42)
Kat	37.63 (1.41)	36.66 (0.22)	37.24 (0.62)	36.41 (0.32)
Kmo	37.75 (0.52)	37.56 (0.99)	37.61 (0.90)	38.96 (0.51)
Kynu	36. 45 (1.02)	36.44 (0.13)	36.98 (0.67)	36.75 (0.95)
Haao	38.71 (0.93)	37.45 (1.21)	38.23 (0.87)	39.26 (0.31)
Qprt	37.44 (0.23)	38.40 (0.26)	38.48 (0.22)	38.94 (0.49)
canx	24.31 (0.21)	24.24 (0.21)	24.29 (0.19)	24.55 (0.23)
atp5b	21.31 (0.21)	21.82 (0.10)	21.38 (0.32)	21.59 (0.13)
eif4a2	22.34 (0.21)	23.34 (0.23)	22.45 (0.16)	23.45 (0.39)

Striatum	WT		R6/2	
	4week	12week	4week	12week
Tdo2	38.94 (0.62)	39.99 (0.33)	38.69 (0.41)	39.46 (0.36)
Ido2	37.01 (0.59)	37.88 (0.21)	37.37 (0.63)	37.52 (0.32)
Kat	38.75 (0.67)	38.62 (0.45)	38.70 (0.66)	38.87 (0.29)
Kmo	38.23 (0.68)	38.54 (1.01)	38.25 (0.50)	38.47 (1.11)
Kynu	37.07 (0.51)	37.30 (0.60)	39.95 (0.36)	38.44 (0.21)
Haao	37.44 (0.39)	36.69 (0.42)	36.72 (0.33)	36.92 (0.41)
Qprt	36.72 (0.33)	37.58 (0.29)	37.18 (0.34)	38.22 (0.53)
canx	24.53 (0.94)	24.01 (1.10)	24.54 (0.92)	24.04 (2.05)
atp5b	21.45 (1.40)	21.75 (0.25)	21.28 (1.50)	21.17 (2.24)
eif4a2	22.83 (0.71)	23.39 (0.94)	22.18 (0.62)	23.69 (1.22)

* Ct values are shown in mean ± SD. Values greater than 36 are in red.

Supplementary Table S2. ***Mean qPCR threshold (C_t) values of reference and target genes in liver from R6/2 and R6/2xKMO cross mice.*** **(Figure 1b)**

	WT		R6/2			R6/2 x KMO (12 weeks)					
	4week	12week	4week	12week		WT:$KMO^{+/+}$	WT: $KMO^{+/-}$	WT: $KMO^{-/-}$	R6/2:$KMO^{+/+}$	R6/2:$KMO^{+/-}$	R6/2:$KMO^{-/-}$
Tdo2	21.52 (0.23)	21.42 (0.32)	21.45 (0.22)	23.22 (0.31)	*Tdo2*	22.08 (0.40)	22.74 (0.11)	21.85 (0.32)	22.44 (0.35)	21.91 (0.31)	21.97 (0.23)
Ido2	25.12 (0.11)	24.96 (0.22)	25.22 (0.29)	25.12 (0.27)	*Ido2*	26.77 (0.38)	26.68 (0.33)	26.62 (0.61)	27.31 (0.22)	26.01 (0.63)	25.97 (0.32)
Kat	24.31 (0.16)	24.61 (0.29)	24.28 (0.21)	24.91 (0.23)	*Kat*	25.49 (0.14)	25.43 (0.23)	25.52 (0.19)	25.43 (0.46)	25.64 (0.32)	25. 54 (0.22)
Kmo	22.32 (0.21)	22.66 (0.19)	22.29 (0.19)	23.82 (0.44)	*Kmo*	25.19 (0.20)	25.17 (0.21)	25.27 (0.14)	25.48 (0.37)	25.86 (0.22)	28.31 (0.19)
Kynu	24.53 (0.18)	24.66 (0.32)	24.52 (0.31)	25.96 (0.29)	*Kynu*	25.64 (0.32)	26.31 (0.22)	25.77 (0.35)	26.52 (0.38)	26.55 (0.32)	26.12 (0.43)
Haao	22.32 (0.19)	22.45 (0.31)	22.38 (0.22)	23.77 (0.23)	*Haao*	22.23 (0.32)	22.23 (0.23)	23.01 (0.35)	23.28 (0.24)	23.11 (0.29)	22.91 (0.23)
Qprt	23.21 (0.21)	23.51 (0.15)	23.49 (0.28)	24.75 (0.28)	*Qprt*	24.29 (0.32)	24.85 (0.21)	24.48 (0.19)	25.02 (0.32)	24.65 (0.23)	24.76 (0.41)
Hprt	26.31 (0.11)	26.96 (0.23)	26.11 (0.22)	26.84 (0.27)	*Hprt*	26.82 (0.27)	26.72 (0.13)	26.85 (0.31)	26.65 (0.28)	26.43 (0.23)	26.32 (0.21)
Canx	24.31 (0.21)	24.24 (0.21)	24.29 (0.19)	24.28 (0.34)	*Canx*	24.17 (0.26)	24.86 (0.21)	24.15 (0.21)	24.75 (0.29)	24.32 (0.25)	24.45 (0.30)
Eif4a2	25.31 (0.13)	24.96 (0.18)	25.11 (0.31)	25.12 (0.24)	*Eif4a2*	25.98 (0.40)	25.61 (0.12)	26.06 (0.41)	25.41 (0.33)	25.43 (0.22)	25.54 (0.12)

* Ct values are shown in mean ± SD.

Supplementary Table S3. ***Mean qPCR threshold (C_t) values of reference and target genes in spleen from the R6/2 and R6/2xKMO cross mice.*** **(Figure 1c)**

	WT		R6/2			R6/2 x KMO (12 weeks)					
	4week	12week	4week	12week		WT:$KMO^{+/+}$	WT: $KMO^{+/-}$	WT: $KMO^{-/-}$	R6/2:$KMO^{+/+}$	R6/2:$KMO^{+/-}$	R6/2:$KMO^{-/-}$
Tdo2	32.33 (0.11)	34.46 (0.31)	32.19 (0.21)	34.12 (0.19)	*Tdo2*	36.25 (0.27)	36.55 (0.33)	37.14 (0.11)	36.02 (0.18)	37.03 (0.32)	36.97 (0.54)
Ido2	32.31 (0.29)	35.80 (0.17)	32.11 (0.19)	35.06 (0.21)	*Ido2*	36.94 (0.32)	36.71 (0.39)	36.92 (0.18)	36.18 (0.21)	36.77 (0.34)	36.73 (0.32)
Kat	33.34 (0.21)	36.91 (0.28)	33.32 (0.24)	36.67 (0.34)	*Kat*	37.08 (0.45)	36.98 (0.45)	31.93 (0.22)	36.57 (0.31)	36.43 (0.23)	32.33 (0.21)
Kmo	23.43 (0.17)	26.41 (0.29)	23.48 (0.25)	25.69 (0.21)	*Kmo*	27.29 (0.28)	27.25 (0.28)	25.02 (0.27)	26.57 (0.26)	27.14 (0.21)	25.22 (0.22)
Kynu	25.43 (0.21)	27.02 (0.22)	25.44 (0.19)	26.79 (0.32)	*Kynu*	27.34 (0.22)	27.97 (0.18)	27.24 (0.32)	27.67 (0.19)	26.22 (0.15)	26.29 (0.13)
Haao	24.24 (0.22)	26.52 (0.15)	24.62 (0.21)	26.02 (0.23)	*Haao*	27.67 (0.32)	26.55 (0.23)	26.69 (0.33)	25.18 (0.27)	26.34 (0.22)	27.92 (0.43)
Qprt	27.28 (0.18)	29.84 (0.22)	27.45 (0.17)	30.11 (0.18)	*Qprt*	30.63 (0.59)	31.23 (0.22)	30.56 (0.41)	31.14 (0.24)	30.22 (0.32)	30.93 (0.65)
Hprt	20.34 (0.21)	26.43 (0.11)	20.45 (0.16)	26.32 (0.18)	*Hprt*	26.31 (0.21)	26.82 (0.10)	26.38 (0.32)	26.59 (0.13)	26.43 (0.23)	26.32 (0.22)
B2m	19.43 (0.18)	20.11 (0.28)	19.32 (0.23)	20.95 (0.17)	*B2m*	20.63 (0.26)	20.51 (0.21)	20.67 (0.29)	20.22 (0.32)	20.01 (0.45)	20.94 (0.45)
Atp5b	25.33 (0.22)	24.43 (0.15)	25.21 (0.19)	24.39 (0.13)	*Atp5b*	25.43 (0.18)	25.69 (0.11)	25.42 (0.21)	25.46 (0.19)	25.65 (0.22)	25.33 (0.23)

* Ct values are shown in mean ± SD. Values greater than 36 are in red.

Supplementary Table S4. ***Mean qPCR threshold (C_t) values of reference and target genes in peritoneal macrophages from the R6/2 and R6/2xKMO cross mice.*** **(Figure 1d)**

	WT		R6/2			R6/2 x KMO (12 weeks)					
	4week	12week	4week	12week		WT:KMO$^{+/+}$	WT: KMO$^{+/-}$	WT: KMO$^{-/-}$	R6/2:KMO$^{+/+}$	R6/2:KMO$^{+/-}$	R6/2:KMO-$^{/-}$
Tdo2	**38.23 (0.87)**	**37.61 (0.90)**	**38.71 (0.93)**	**37.56 (0.99)**	***Tdo2***	**35.20 (0.59)**	**38.93 (1.21)**	**37.68 (0.41)**	**38.50 (1.11)**	**39.01 (0.22)**	**38.34 (0.91)**
Ido2	**37.44 (0.23)**	**39.26 (0.31)**	**37.45 (1.21)**	**38.96 (0.51)**	***Ido2***	**38.50 (0.91)**	**38.45 (2.33)**	**38.17 (1.31)**	**38.87 (1.64)**	**38.54 (0.78)**	**38.65 (0.69)**
Kat	**36.98 (0.67)**	**38.40 (0.26)**	**36. 45 (1.02)**	**38.94 (0.49)**	***Kat***	**37.94 (0.97)**	**38.52 (0.63)**	**37.69 (0.69)**	**37.93 (0.53)**	**38.86 (0.43)**	**39.11 (1.08)**
Kmo	34.75 (0.32)	35.45 (0.87)	34.82 (0.51)	35.39 (0.53)	***Kmo***	31.78 (1.63)	33.87 (1.72)	**36.28 (0.74)**	31.14 (1.01)	33.23 (0.45)	**36.11 (0.32)**
Kynu	35.86 (0.29)	35.12 (0.65)	35.44 (0.82)	34.75 (0.95)	***Kynu***	35.46 (0.87)	35.94 (1.96)	35.36 (1.06)	30.88 (1.38)	33.43 (0.45)	35. 32 (0.23)
Haao	32.76 (0.67)	34.43 (0.57)	33.02 (0.70)	33.20 (0.32)	***Haao***	34.59 (0.76)	35.34 (2.18)	34.68 (2.12)	33.03 (1.31)	34.23 (0.34)	24.93 (0.22)
Qprt	35.77 (0.43)	35.44 (0.34)	35.43 (0.22)	35.33 (0.54)	***Qprt***	35.66 (0.32)	34.98 (0.56)	35.34 (0.32)	35.98 (0.44)	34.33 (0.54)	34.23 (0.66)
Atp5b	25.43(0.22)	27.63 (0.94)	25.94 (0.45)	27.49 (0.51)	***Atp5b***	27.58 (0.88)	27.01 (0.98)	27.34 (0.22)	26.97 (0.18)	27.24 (0.32)	26.67(0.19)
Gapdh	25.74 (0.68)	26.23 (0.78)	25.65 (0.74)	26.39 (0.48)	***Gapdh***	26.91 (0.75)	27.23 (0.81)	27.16 (1.20)	27.15 (1.52)	26.85 (0.98)	27.32 (1.01)
Actb	23.98 (0.22)	24.39 (0.70)	24.01 (0.43)	24.10 (0.48)	***Actb***	24.44 (0.85)	23.46 (0.98)	24.65 (1.01)	24.30 (1.73)	24.55 (0.23)	24.98 (0.56)

* Ct values are shown in mean ± SD. Values greater than 36 are in red.

Supplementary Table S5. Real-time quantitative qPCR assays

Transcript/ Gene Symbol	Transcript/Gene name	Taqman Assay ID	Exon boundary
Tdo2	Tryptophan 2,3-Dioxygenase	Mm00451269_m1	Exon 8 - 9
Ido2	Indoleamine 2,3-dioxygenase	Mm00524210_m1	Exon 9 - 10
Aadat	Aminoadipate aminotransferase	Mm00496169_m1	Exon 10 - 11
Kynu	Kynureninase	Mm00551012_m1	Exon 2 - 3
Kmo	Kynurenine 3-Monooxygenase	Mm01321341_m1	Exon 3 - 4
Haao	3-Hydroxyanthranilate 3,4-Dioxygenase	Mm005117945_m1	Exon 1 - 2
Qprt	Quinolinate Phosphoribosyltransferase	Mm00504998_m1	Exon 2 - 3
Canx	Calnexin	Mm00500330_m1	Exon 4 - 5
B2m	Beta-2-microglobulin	Mm00437762_m1	Exon 1 -2
Atp5b	ATP synthase subunit Beta	Mm00443967	Exon 4 - 5
Hprt	Hypoxanthine-guanine phosphoribosyltransferase	Mm03024075_m1	Exon 2 - 3
Eif4a2	Eukaryotic translation initiation factor 4A2	Mm01730183_gH	Exon 7 - 8

Supplementary Table S6. Summary of antibodies

Name of antibody	Species	Concentration	Source/catalogue number
**Western blot**			
**KMO**	mouse	1:5000	Abcam/ab167274
**HSP90**	mouse	1:1000	Santa-cruz/Sc-13119
**Anti - rabbit**	donkey	1:1000	Dako/SA1-200
**Immunohistochemistry**			
**F4/80**	rat	1:500	Abcam/ab6640
**Alexa Fluor 488**	donkey	1:500	Vector Labs/PO488

Supplementary Table S7. *t*-test statistics for gene expression (qPCR) (Figure 1)

Liver transcripts	4 weeks	12 weeks
Tdo2	t(13.21) = 1.298, *p* = 0.216	t(11) = 3.207, *p* = 0.008
Ido2	t(27) = 1.198, *p* = 0.241	t(12) = 1.1027 *p* = 0.324
Kat	t(15.87) = 0.959, *p* = 0.351	t(11) = 1.290 *p* = 0.223
Kynu	t(12) = 3.264, *p* = 0.994	t(12) = 2.682, *p* = 0.019
Kmo	t(25) = 0.419, *p* = 0.678	t(12) = 3.246, *p* = 0.007
Haao	t(12) = 3.264, *p* = 0.383	t(12) = 2.815, *p* = 0.015
Qprt	t(26) = 0.698, *p* = 0.491	t(12) = 3.264, *p* = 0.006
**Spleen transcripts**		
Kat	t(25) = 1.537, *p* = 0.137	t11) = 0.01315, *p* = 0.989
Kynu	t(24) = 1.826, *p* = 0.080	t(13) = 2.165, *p* = 0.049
Kmo	t(24) = 0.08277, *p* = 0.934	t(13) = 5.604, *p* < 0.001
Haao	t(25) = 0.1035, *p* = 0.918	t(13) = 4.120, *p* = 0.001
Qprt	t(25) = 0.650, *p* = 0.5213	t(12) = 3.127, *p* = 0.008
**pMO transcripts**		
Qprt	t(24)=0.9340, *p* = 0.359	t(12) = 1.069, *p* = 0.306
Haao	t(25) = 0.2288, *p* = 0.820	t(11) = 2.207, *p* = 0.049
Kmo	t(26) = 1.080, *p* = 0.290	t(12) = 2.557, *p* = 0.025
Kynu	t(23) = 0.224, *p* = 0.8246	t(6.97) = 2.751, *p* = 0.028

Supplementary Table S8. One-way ANOVA and *t*-test statistics for protein expression (Western blot) (Figure 2c and e)

Liver (c)	12 weeks
KMO	F(5,20) = 28.04, *p* < 0.001
**Liver (e)**	
KMO	t(14) = 4.589, *p* = 0.004

Supplementary Table S9. One-way ANOVA test statistics for gene expression (qPCR) (Figure 3)

Liver transcripts	12 weeks
Tdo2	$F(5,39) = 2.754$, $p = 0.0318$
Ido2	$F(5,39) = 21.28$, $p < 0.001$
Kat	$F(5,39) = 3.105$, $p = 0.0187$
Kynu	$F(5,37) = 4.052$, $p = 0.004$
Kmo	$F(5,39) = 56.53$, $p < 0.001$
Haao	$F(5,37) = 3.569$, $p = 0.009$
Qprt	$F(5,39) = 0.896$, $p = 0.4932$
**Spleen transcripts**	
Kat	$F(5,39) = 20.33$, $p < 0.001$
Kynu	$F(5,36) = 2.628$. $p = 0.039$
Kmo	$F(5,39) = 20.15$, $p < 0.001$
Haao	$F(5,36) = 8.180$, $p < 0.001$
Qprt	$F(5,38) = 6.055$, $p < 0.001$
**pMO transcripts**	
Qprt	$F(5,38) = 4.103$, $p = 0.004$
Haao	$F(5,37) = 3.264$, $p = 0.0153$
Kynu	$F(5,37) = 22.98$, $p < 0.001$
Kmo	$F(5,40) = 194.4$, $P < 0.001$

Supplementary Table S10. One-way ANOVA test statistics for peripheral metabolites (HPLC) (Figure 4)

Liver	12 weeks
Tryptophan	$F(5,30) = 0.5361$, $p = 0.7473$
Kynurenine	$F(5,29) = 20.75$, $p < 0.001$
Anthranilic acid	$F(5,30) = 21.23$, $p < 0.001$
Kynurenic acid	$F(5,29) = 48.92$, $p < 0.001$
3 - Hydroxykynurenine	$F(5,30) = 10.75$, $p < 0.001$
Quinolinic acid	Not obtainable
**Spleen**	
Tryptophan	$F(5,30) = 0.331$, $p = 0.889$
Kynurenine	$F(5,29) = 41.16$, $p < 0.001$
Anthranilic acid	$F(5,28) = 57.55$, $p < 0.001$
Kynurenic acid	$F(3,17) = 32.\ 48$, $p < 0.001$
3 - Hydroxykynurenine	$F(5,28) = 21.70$, $P < 0.001$
Quinolinic acid	$F(5,27) = 40.59$, $p < 0.001$

Supplementary Table S11. One-way ANOVA test statistics for brain metabolites (HPLC) (Figure 5)

**Cortex**	**12 weeks**
Tryptophan	$F(5,27) = 4.350$, $p = 0.004$
Kynurenine	$F(5,29) = 34.89$, $p < 0.001$
Anthranilic acid	$F(5,29) = 14.86$, $p < 0.001$
Kynurenic acid	$F(5,20) = 8.202$, $p < 0.001$
3 - Hydroxykynurenine	$F(5,29) = 12.07$, $p < 0.001$
Quinolinic acid	Not obtainable
**Striatum**	
Tryptophan	$F(5,30) = 1.183$, $p < 0.3408$
Kynurenine	$F(5,30) =16.96$, $p < 0.001$
Anthranilic acid	$F(2,9) = 3.789$, $p = 0.064$
Kynurenic acid	$F(5,26) = 14.33$, $p < 0.001$
3 - Hydroxykynurenine	$F(5,25) = 7.998$, $p < 0.001$
Quinolinic acid	$F(5,25) = 1.504$, $p = 0.224$
**Cerebellum**	
Tryptophan	$F(5,30) = 0.426$, $p = 0.826$
Kynurenine	$F(5,28) = 25.44$, $p < 0.001$
Anthranilic acid	$F(5,27) = 24.58$, $p < 0.001$
Kynurenic acid	$F(5,25) = 9.00$, $p < 0.001$
3 - Hydroxykynurenine	$F(5,27) = 14.97$, $p < 0.001$
Quinolinic acid	Not obtainable

Supplementary Table S12. One-way ANOVA test statistics for cytokine levels (MSD) (Figure 6)

**Cytokines**	**12 weeks**
TNF-α	$F(5,18) = 19.55$, $p < 0.001$
IL-1β	$F(5,18) = 5.657$, $p = 00026$
IL-2	$F(5,18) = 1.422$, $p = 0.2638$
IL - 4	$F(5,18) = 19.00$, $p < 0.001$
IL-5	$F(5,18) = 9.258$, $p < 0.001$
IL-6	$F(5,18) = 16.58$, $p < 0.001$
IL-10	$F(5,18) = 2.089$, $p = 0.1140$
IFN - gamma	$F(5,18) = 1.328$, $p = 0.2969$

Supplementary Table S13 One-way ANOVA test statistics for F4/80 fluorescent expression (Figure 7)

**Liver**	**12 weeks**
F4/80	$F(5,17) = 29.114$, $p < 0.001$
